# Comparative genomics and phenotypic divergence of ERIC I and ERIC II genotypes of *Paenibacillus larvae*, the causative agent of American Foulbrood disease

**DOI:** 10.1371/journal.ppat.1014507

**Published:** 2026-08-13

**Authors:** Md. Mubarack Hossain, Kaitlyn Coleman, Matej Medvecky, Matthew Moore, Xavier Didelot, David Chandler, Christopher D. A. Rodrigues

**Affiliations:** 1 School of Life Sciences, University of Warwick, Coventry, United Kingdom; 2 Bioinformatics and Digital Health Services, Research Technology Platforms, University of Warwick, Coventry, United Kingdom; University of Toronto, CANADA

## Abstract

Honeybees of the species *Apis mellifera* are important pollinators of crops and wild plants. *Paenibacillus larvae*, a spore-forming bacterium, is a problematic pathogen that causes American foulbrood (AFB) in honeybee larvae worldwide. In many countries, AFB is a notifiable disease, requiring the destruction of diseased colonies, resulting in economic loss that impacts beekeeping and agriculture. Disease onset starts with larval ingestion of *P. larvae* spores, which germinate into growing cells that proliferate in the larval gut, leading to larval death and eventually bee colony collapse. As infection progresses, *P. larvae* produce spores, reinitiating the disease cycle. Thus, growth, sporulation and germination underlie AFB. In this study, using various microbiological assays, quantitative cell biology methods, transmission electron microscopy and genomics, we sought to identify genetic and phenotypic characteristics associated with the predominant ERIC I and ERIC II genotypes of *P. larvae* during growth, sporulation and germination. Extending previous findings, our data identify genetic differences between ERIC I and ERIC II strains and some genetic variation between strains of the same ERIC type. Furthermore, we describe significant differences in cellular morphology during growth, differences in spore envelope structure and differences in germination efficiency between ERIC I and ERIC II genotypes. Collectively, our findings improve understanding of *P. larvae* biology and provide a foundation for developing genotype-specific disease management strategies for AFB.

## Author summary

Honeybees (*Apis mellifera*) are essential pollinators that support global agriculture and ecosystem stability. American foulbrood (AFB) is a highly destructive disease of honeybee larvae caused by the spore-forming bacterium *Paenibacillus larvae*. Infection occurs when larvae ingest bacterial spores, which germinate in the gut, proliferate, and ultimately cause larval death, leading to colony losses. Despite the genetic diversity of *P. larvae*, the biological differences between the major disease-associated genotypes remain poorly understood. Here, we compared isolates belonging to the predominant ERIC I and ERIC II genotypes and examined traits important for bacterial survival and disease progression, including growth, sporulation, and spore germination. We identified genotype-specific differences in growth dynamics, cellular morphology, spore structure, and germination behaviour. These findings provide new insight into the biology of *P. larvae* and suggest that variation between genotypes may contribute to differences in disease dynamics. This study provides a foundation for future investigations into genotype-specific pathogenic mechanisms and may facilitate the development of targeted strategies for the prevention and control of American foulbrood.

## Introduction

Pollination is essential for sustaining ecosystem balance and supporting crop production. The European honeybee (*Apis mellifera*) is an important pollinator species globally, aiding in the pollination of approximately 90–130 commercial crops that are vital to the human diet [1]. These crops contribute significantly to global agricultural output and nutritional security. Modern agricultural practices, which utilise a high density of plants per acre, increase the demand for pollination services, making honeybee health critical not only for apiculture but also for agriculture and global food security [2]. However, honeybee populations have been declining at an alarming rate due to multiple factors, including habitat loss, pesticide exposure, nutritional stress, and pathogens [3]. Among these stressors, infectious diseases pose a serious threat to colony survival, particularly American foulbrood (AFB), the most destructive brood disease of honeybees worldwide, caused by *Paenibacillus larvae*, a Gram-positive, spore-forming bacterium [4].

AFB infects first or second-instar larvae (within 48 hours of hatching) when they consume food contaminated with *P. larvae* spores [5]. Although these spores are present throughout a contaminated hive, they germinate massively in the gut of bee larvae approximately 12 hours after ingestion [6,7]. This early susceptibility of larvae makes disease prevention particularly challenging [8]. Following germination of spores in the larval midgut, *P. larvae* vegetative cells then proliferate at this location and eventually breach the midgut epithelium, invading the haemocoel and causing septicaemia [9]. The bacteria then release various virulence-associated factors, including enzymes such as proteases, collagenases, and chitinases, which break down the host tissues, leading to larval death within 3–12 days [10]. As infection progresses and larvae die, the depletion in nutrient levels causes *P. larvae* cells to stop multiplying and develop into spores, resulting in millions of spores contained within the dead remains of each larva [11]. These highly resilient spores can persist in the environment for decades and spread easily between bee colonies. Consequently, spores can spread between bee colonies, leading to the infection of neighbouring hives [12], which sparks outbreaks that cause substantial economic loss for beekeepers and threaten both honey production and crop pollination services [13].

*P. larvae* is known to be an extremely variable pathogen, and through ERIC (Enterobacterial Repetitive Intergenic Consensus) typing, different *P. larvae* genotypes have been identified, namely ERIC I, II, III, IV and V [14,15]. Among these, ERIC I is the most widespread genotype and is generally regarded as less virulent than ERIC II [16]. ERIC I strains typically take longer to kill infected larvae, allowing infected larvae to survive until cell capping, which reduces the efficiency of hygienic removal by nurse bees; as a result, infected larvae remain within capped cells, allowing *P. larvae* to complete sporulation and produce high numbers of infectious spores, and contribute to disease progression within and between colonies [14]. Conversely, ERIC II is less common but is known for its higher virulence, which manifests as faster killing of infected larvae compared to ERIC I [9,10]. This results in the accumulation of infected and diseased brood before effective hygienic removal can occur, leading to accelerated brood loss and earlier colony collapse [17]. Despite these notable differences between ERIC I and ERIC II in AFB disease progression, and the known impact of *P. larvae* in honeybee health, there has been limited research into the *P. larvae* strain-specific differences in growth, sporulation, germination, and genome variability.

Given the significant impact of *P. larvae* on honeybee health and the distinct differences observed between ERIC I and ERIC II strains, and the need for development of approaches that distinguish ERIC I and ERIC II isolates, in this study we aimed to identify genetic and phenotypic characteristics associated with the predominant ERIC I and ERIC II genotypes of *P. larvae.* We conducted a comprehensive characterisation comparing the genome, as well as the growth, sporulation, and germination phenotypes, of six different *P. larvae* strains isolated from diverse geographical locations. We focused our characterisation on three ERIC I and three ERIC II strains to identify shared and distinguishing features of ERIC I and ERIC II types and to determine the extent of strain-specific variability within each ERIC type. Collectively, our examination of strain-specific growth, sporulation, germination, and genomic variability contributes to a deeper understanding of the genetic, growth, developmental, and morphological variation among *P. larvae* strains and provides a basis for the development of targeted, genotype-specific disease management strategies for AFB.

## Results

### Genomic characterisation of ERIC I and ERIC II strains

We selected six strains, three ERIC I and three ERIC II, originating from various locations in Europe (Table 1). To begin the characterisation of these strains, we conducted whole-genome sequencing (WGS). Consistent with the previous report showing that ERIC I genomes are larger than ERIC II genomes [10]. ERIC I strains (7841, 7845, and 7856) showed genome sizes ranging from 4.10 to 4.16 Mb with GC contents of approximately 44.0%. In contrast, ERIC II strains (7842, 7851, and 7853) had smaller genome sizes, ranging from 3.69 to 3.74 Mb, and slightly higher GC contents of approximately 44.5%. Assembly metrics also differed between the two groups, with ERIC I strains comprising 134–178 contigs and N50 values ranging from 61,840–80,118 bp, whereas ERIC II strains contained 297–311 contigs and showed lower N50 values ranging from 25,137–25,910 bp.

**Table 1:** *Paenibacillus larvae* strains used in this study.

Strains	Genotype	Country of Origin	Continent
7841	ERIC-I	Germany	Europe
7845	ERIC-I	Czech Republic	Europe
7856	ERIC-I	United Kingdom	Europe
7842	ERIC-II	Germany	Europe
7851	ERIC-II	Finland	Europe
7853	ERIC-II	Germany	Europe

**Fig 1:**
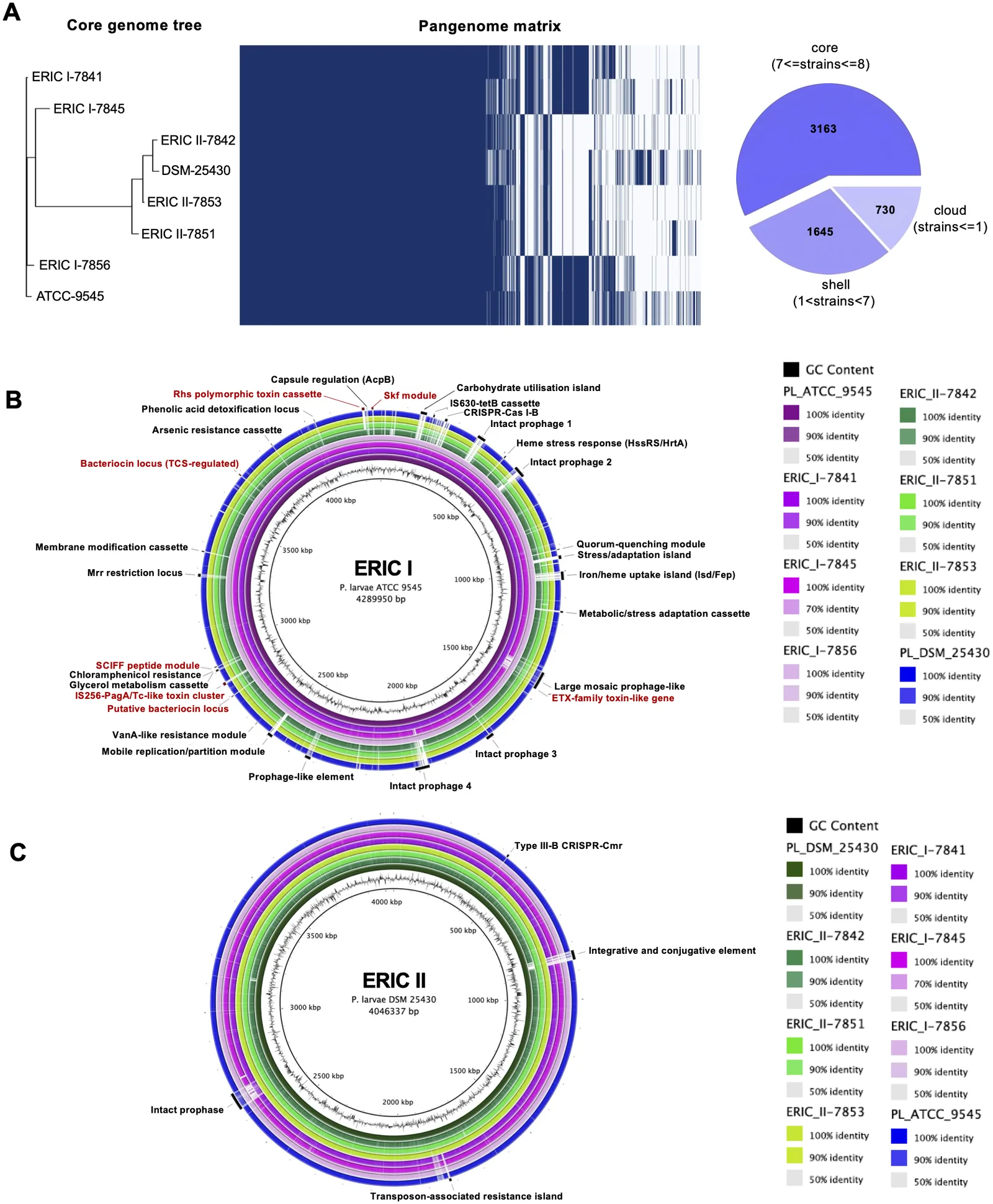
Comparative genomic analysis and pangenome structure of *Paenibacillus larvae* ERIC I and ERIC II strains. In panel **(A)** Pangenome analysis of the studied strains along with two reference strains. The left panel shows a core genome-based phylogenetic tree. The middle section presents the gene presence-absence matrix generated using Roary. The dark blue indicates gene presence and white indicates gene absence across the strains, and the pie chart summarises pangenome composition, showing the number of core-, shell-, and cloud-genome gene clusters. **(B&C)** Whole-genome comparisons of eight *P. larvae* strains, comprising four ERIC I and four ERIC II isolates were performed using the BLAST Ring Image Generator (BRIG). In panel **(B)**, genomes were aligned against the ERIC I reference strain ATCC 9545; in panel **(C)**, genomes were aligned against the ERIC II reference strain DSM 25430. Concentric rings represent individual query genomes relative to the respective reference sequence, with shading intensity corresponding to sequence similarity. Regions lacking alignment indicate genomic divergence, while the outermost ring highlights annotated genomic features that differ between ERIC I and ERIC II.

Pangenome analysis of our six selected *P. larvae* strains, along with ERIC I and ERIC II reference sequences obtained from NCBI database, revealed a large, conserved core genome alongside a smaller, variable accessory genome (Fig 1A). Gene clusters were classified into core (present in>= 7 strains), shell (present in 2–6 strains), and cloud (present in a single strain) based on their presence across the analysed strains. The core genome comprised 3,163 genes, the shell genome included 1,645 genes, and the cloud genome consisted of 730 gene clusters. The gene presence-absence matrix demonstrated genotype-associated differences in accessory gene composition, with strains from the same ERIC type showing more similar gene content profiles (Fig 1A). Phylogenetic analysis based on the shared core-genome further supported the separation between ERIC I and ERIC II lineages. Notably, strain 7842 clustered most closely with the ERIC II reference strain DSM 25430, indicating that these two share the highest genomic similarity among the analysed ERIC II strains (Fig 1A).

To further assess genomic differences between and within the two ERIC types, we conducted a comparative analysis of the predicted proteome of the six newly sequenced strains. Protein clustering analysis across our cohort revealed significant differences in their protein content, cluster formation, and singleton (proteins unique to each strain) (S1B Fig and S1 Table). As expected, the ERIC I strains exhibited higher protein counts than the ERIC II strains, with ERIC I strain 7856 showing the highest number of proteins (5,012 proteins; 4,913 clusters) and ERIC II strain 7842 the lowest (4,317 proteins; 4,226 clusters) (S1B Fig and S1 Table). Across all strains, 3,671 proteins were shared, with 725 exclusive to ERIC I and 241 exclusive to ERIC II. Notably, ERIC I strain 7845 had the highest number of singletons (144; S1 Table), indicating a wide range of unique proteins, whereas ERIC II strain 7853 had the fewest singletons (26; S1 Table). These results support the genetic distinction between ERIC I and ERIC II strains.

Moreover, to evaluate both homology and genetic diversity among the strains in detail, we conducted a thorough whole-genome comparative analysis of our cohort of *P. larvae* strains against two reference strains, ERIC I-ATCC 9545 and ERIC II-DSM25430 (Fig 1B and 1C). BRIG-based whole genome comparison of the ERIC I and the ERIC II strains identified multiple lineage-specific accessory regions distributed across the chromosomes. Three out of four ERIC II samples contain a complete integrative and conjugative element (ICE) comprising a tyrosine recombinase integrase, a putative relaxase (*NicK*), conjugative transfer proteins, secretion-associated components and regulatory genes, consistent with a functional integration-excision and transfer module. This ICE was absent from all analysed ERIC I genomes as well as ERIC II-7842 (Fig 1B and 1C). Both ERIC genotypes harbour prophage derived regions; however, prophage content differs markedly between lineages. ERIC I strains contain four lineage-specific intact prophages and two additional prophage-like regions, including one large mosaic element (Fig 1B). In contrast, ERIC II strains lack the expanded prophage repertoire observed in ERIC I genomes and harbour only a single unique intact prophage that is completely absent in ERIC I-7845 and partially absent in the remaining ERIC I genomes under investigation (Fig 1B and 1C).

Both genotypes encode a Type III-B Clustered Regularly Interspaced Short Palindromic Repeats (CRISPR)-CRISPR-associated (Cas) module. However, ERIC II genomes along with ERIC I-7845, harbour a more complete CRISPR module RAMP (Cmr)-associated cluster including Cmr1, Cas10/Cmr2, Cmr3, Cmr4, Cmr5 and Cmr6, whereas the remaining ERIC I samples retain only Cmr1, Cas10/Cmr2 and Cmr3. In addition, ERIC I genomes carry a complete Type I-B CRISPR-Cas system and an *mrr* restriction locus, neither of which was identified in ERIC II strains (Fig 1B and 1C).

Several antibiotic resistance-associated loci were specific to ERIC I genomes. One notable region comprises an IS630-associated *tetB*-like cassette, encoding a major facilitator superfamily (MFS) transporter (100% coverage, 58.2% amino acid identity to UniProtKB homologues). The locus is flanked by a transposase and a split putative ABC transporter showing 100% coverage and 85% and 59% identity to homologues from *Paenibacillus riograndensis* and *Paenibacillus graminis*, respectively.

A catB-like chloramphenicol resistance gene was identified in ERIC I genomes, encoding a chloramphenicol acetyltransferase with 100% coverage and 97.2% amino acid identity to a homologue from *Bacillus salipaludis*. A vanA-like determinant, annotated by Comprehensive Antibiotic Resistance Database (CARD) as vanF (100% coverage, 97.9% identity), was detected in all ERIC isolates except ERIC II strain 7851 and is associated with reduced glycopeptide resistance in *Paenibacillus popilliae* via D-Ala–D-Lac peptidoglycan synthesis.

ERIC I genomes also carried an arsenic resistance cassette together with additional detoxification loci, including a phenolic acid decarboxylase system and genes involved in membrane stress responses and efflux mechanisms.

ERIC II genomes, by comparison, carried a transposon-associated resistance island containing an additional copy of *sugE* together with *ykkD*, as annotated by Prokka. BLASTP showed SugE is homologous to the guanidinium efflux protein GdnC from *Bacillus licheniformis* (93% coverage, 43.4% identity), while YkkD is similar to GdnD from *Bacillus subtilis* (100% coverage, 32.7% identity). UniProtKB further identified YkkD as a QacE-family small multidrug resistance transporter, with 100% identity to *P. larvae* and 84.6% identity to *Paenibacillus agilis*. These proteins likely form membrane efflux systems contributing to tolerance of quaternary ammonium compounds and other toxic metabolites.

ERIC I genomes possess an expanded repertoire of toxin- and competition-related regions, including an IS256-associated *pagA*/Tc-like toxin cluster, an epsilon toxin (ETX)- family-like gene, a large Rhs polymorphic toxin cassette, and two bacteriocin loci, one of which is associated with a two-component regulatory system. In addition, a Six Cysteines in Forty-Five Residues (SCIFF) peptide module and a *skf*-associated cluster further contribute to the competition related gene repertoire. These loci were consistently detected across all ERIC I genomes.

Finally, ERIC I genomes contain several lineage specific metabolic and surface-associated regions, including a carbohydrate utilisation and glycosyltransferase-enriched island, an iron/heme uptake locus (*isd*/*fep*), a heme stress response system (HssRS/*hrtA*), membrane modification cassettes and additional stress/adaptation modules. Collectively, these regions contribute to a broader repertoire of accessory genes in ERIC I genomes relative to ERIC II, highlighting the genetic diversity between strains within the same ERIC type.

### Phylogenetic contextualisation of ERIC I and ERIC II strains

To explore the evolutionary relationship among the selected ERIC I and ERIC II *P. larvae* isolates, we performed a phylogenetic analysis using a total of 782 publicly available *P. larvae* genomes along with our six newly sequenced genomes. The resulting phylogeny revealed two distinct and well-separated clades corresponding to the established ERIC I and ERIC II genotypes. Within the ERIC I lineage, isolates were assigned mostly to MLST sequence types ST2, ST5 and ST18, while ERIC II isolates were primarily associated with ST10 and ST11 (S1A Fig). The clustering pattern was consistent across both typing schemes, demonstrating a strong concordance between MLST classification, ERIC genotype, and whole-genome phylogenetic relations. The phylogeny revealed that the six isolates sequenced in this study were distributed across multiple sub-clades within the ERIC I and ERIC II lineages rather than forming a closely related cluster. Median patristic distances between the selected isolates and all isolates of the corresponding lineage exceeded within-lineage distances across the full phylogeny (ERIC I: 3.7 × 10^−4^ vs 2.7 × 10^−4^ substitutions per site; ERIC II: 9.3 × 10^−5^ vs 7.2 × 10^−5^ substitutions per site), demonstrating that the selected isolates are genetically diverse members of both major *P. larvae* lineages. Consistent with the phylogenetic structure, ERIC I isolates exhibited greater diversity than ERIC II isolates, with median within-lineage patristic distances approximately 3.8-fold higher across the full dataset. Overall, these findings indicate that the *P. larvae* species has a clear population structure composed of two evolutionarily stable lineages.

### Growth characteristics of ERIC I and ERIC II strains

**Fig 2:**
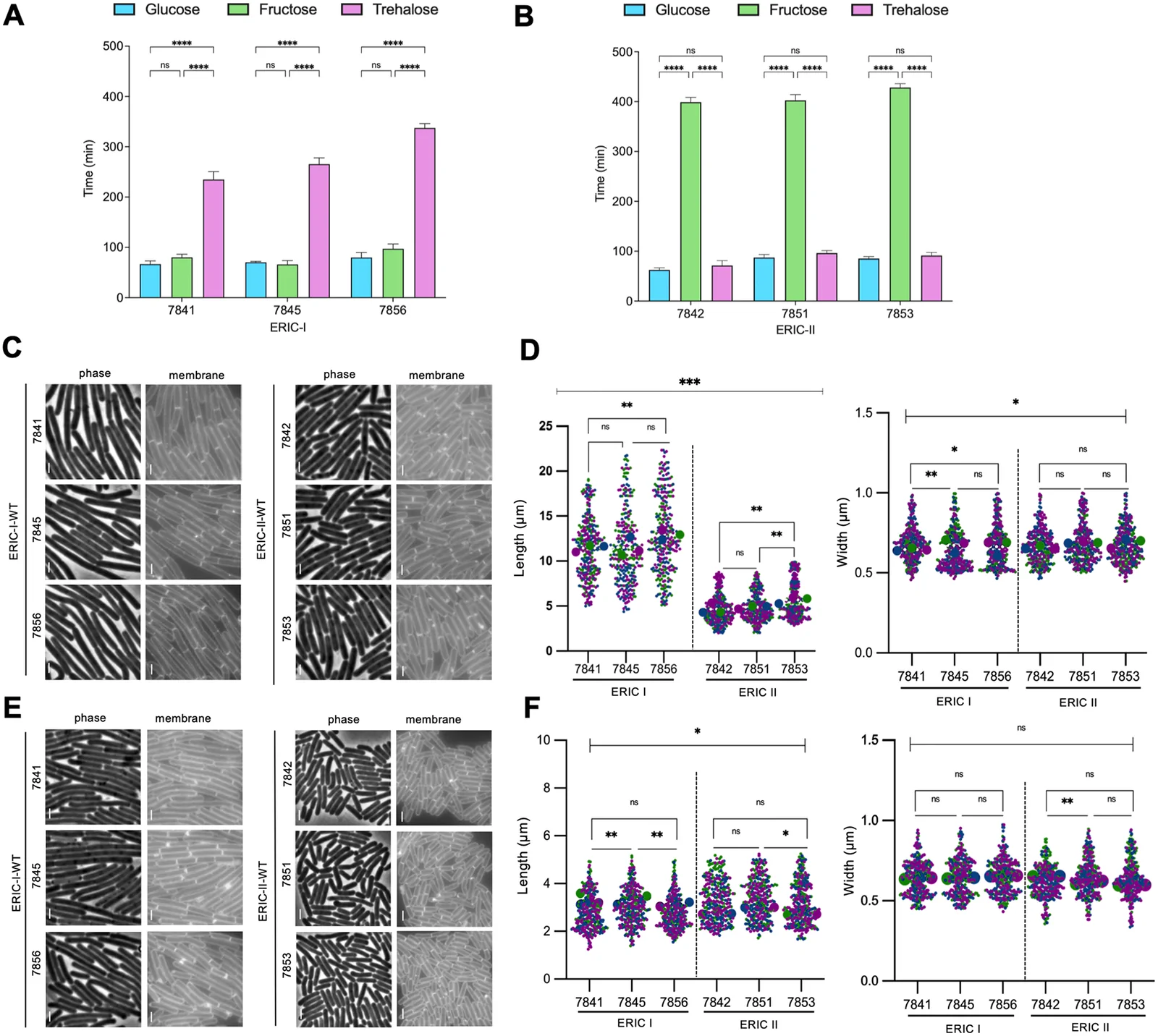
Growth rate and cell morphology of ERIC I and ERIC II strains. **(A & B)** Generation times of ERIC I **(A)** and **(B)** ERIC II strains in MYPGP media supplemented with different sugars. Data are shown as (mean ± SD) of three independent biological replicates. **(C)** Representative micrographs of ERIC I and ERIC II strains during the log-phase in MYPGP media supplemented with 10% glucose. Scale bar, 2 µm. **(D)** Superplots displaying measurements of single-cell length and width for ERIC I and ERIC II strains in the log-phase grown in MYPGP media supplemented with 10% glucose. **(E)** Representative micrographs of ERIC I and ERIC II strains during the stationary phase in MYPGP media supplemented with 10% glucose. Scale bar, 2 µm. **(F)** Superplots displaying measurements of single-cell length and width for ERIC I and ERIC II strains in the stationary phase grown in MYPGP media supplemented with 10% glucose. The data presented in panels D and F were obtained from three independent biological replicates, with 100 cells per replicate. Individual data points represent single cells, and colours denote different replicates. Statistical significance of differences in length and width among strains within each ERIC group was assessed using the Kruskal-Wallis test followed by Dunn’s multiple comparisons test. Differences between ERIC groups were evaluated using the Mann-Whitney U test. Significance levels are indicated as follows: p* < 0.05, p** < 0.01, p*** < 0.001, p**** < 0.0001; ns, not significant.

Our WGS data suggest significant genetic differences between ERIC I and ERIC II strains. Thus, it seemed possible that these differences could impart different growth characteristics to each strain. To test this, we first examined the growth rates of the ERIC I and ERIC II strains in MYPGP media supplemented with different sugars (Glucose, Fructose, and Trehalose) and calculated the generation times (Figs 2A, 2B and S2). In MYPGP supplemented with glucose, the ERIC I and ERIC II strains exhibited similar generation times that varied between 60 and 80 minutes (Fig 2A and 2B). In MYPGP supplemented with fructose, we observed that ERIC I strains exhibited a shorter generation time compared to ERIC II strains (Fig 2A). Thus, ERIC I strains are better adapted to growth in fructose than ERIC II strains. Interestingly, the opposite was observed for growth in MYPGP supplemented with trehalose: the ERIC II strains exhibited a shorter generation time, compared to ERIC I strains (Fig 2B). Thus, ERIC II strains are better adapted to growth in trehalose than ERIC I strains.

### Morphological characteristics of ERIC I and ERIC II strains

Previous works suggest that ERIC I and ERIC II strains exhibit differences in cellular morphology, with the ERIC I strain ATCC 9545 producing longer cells and the ERIC II strain 04–309 producing shorter cells during the log phase of growth [18]. However, it remains unclear whether different ERIC I and ERIC II strains exhibit similar morphological characteristics and whether morphological differences exist within the same ERIC type. To answer this question, we examined the cellular morphology of our six strains during the log phase and the stationary phase, in liquid MYPGP media supplemented with glucose. As expected, both ERIC I and ERIC II strains exhibited a rod-shaped morphology during the exponential and stationary phases. Consistent with previous work [18], we observed that all three ERIC I strains produced longer rod-shaped cells than the three ERIC II strains during the log-phase (Fig 2C) and in both the ERIC I and ERIC II strains, the rod-shaped cells were visibly shorter during the stationary phase, compared to log-phase (Fig 2E).

To quantitatively confirm these observations, we performed image segmentation analysis to measure the length and width of these six strains at both growth stages. Consistent with our qualitative observations, ERIC I strains measured approximately 18–23 µm in length during log-phase growth and were at least threefold longer than ERIC II strains, which measured approximately 5–8 µm (Fig 2D). Notably, despite this marked morphological difference, comparative genomic analysis showed no major differences in their division and cell wall cluster (DCW) genes, which were found to be highly conserved between the two groups (S1C Fig).

In previous work, a genetically modified ERIC I strain (ATCC 9545) and ERIC II strain (04-309) of *P. larvae* expressing plasmid-encoded GFP (plasmid pAD43‐25) was used to examine cellular morphology [18]. Since expression of fluorescent proteins from plasmids is known to cause varying levels of toxicity [19], we investigated whether pAD43-25 affects cellular morphology, we introduced the plasmid into our ERIC I and ERIC II strains and compared the cell length and width of plasmid-carrying strains with those of the corresponding strains lacking the plasmid. As expected, the three ERIC I strains were consistently longer than the three ERIC II strains during both the logarithmic and stationary phases of growth (S3A and S3C Fig). However, quantitative analysis suggests that the addition of the plasmid to ERIC I or ERIC II strains results in a significant increase in cell length (S3B and S3D Fig). Moreover, comparison of the transformed and non-transformed strains showed that the presence of the plasmid resulted in a significant increase in cell length in both ERIC I and ERIC II strains during logarithmic growth (S3E Fig). We also found that during the stationary phase, cell length increased significantly in ERIC I strains carrying the plasmid, whereas no significant differences were observed in ERIC II strains compared with their plasmid-free counterparts (S3F Fig). Based on these findings, we conclude that genetic manipulation of *P. larvae* with pAD43‐25 significantly alters their cellular morphology.

### Sporulation efficiency of ERIC I and ERIC II strains

**Fig 3:**
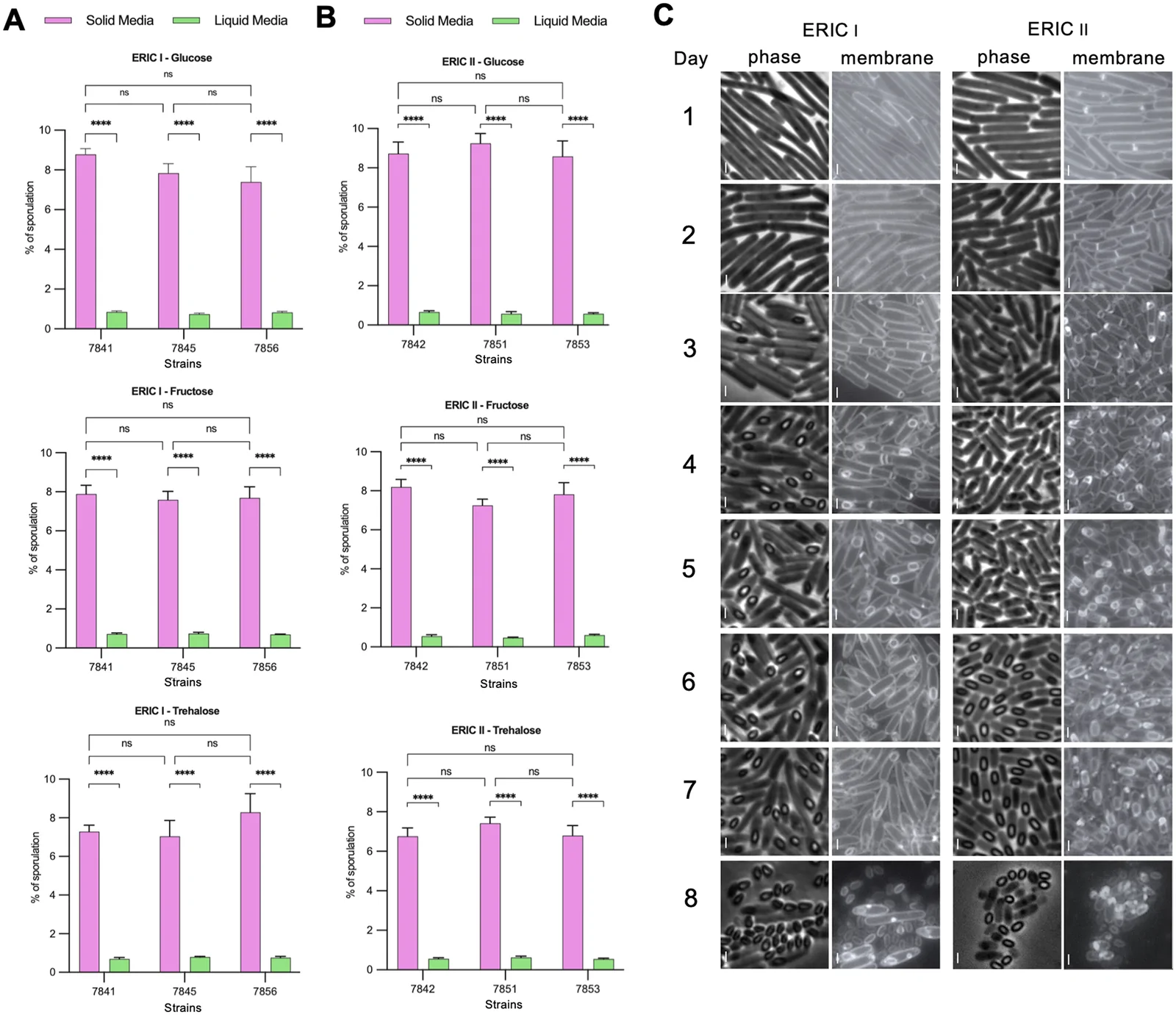
Sporulation efficiency and morphological stages of sporulation in ERIC I and ERIC II strains. **(A & B)** Sporulation efficiencies of **(A)** ERIC I and **(B)** ERIC II strains grown in MYPGP media supplemented with 20% glucose, fructose, or trehalose, in liquid and solid MYPGP media. Data are presented as (mean ± SD) of three independent biological replicates. **(C)** Representative micrographs showing morphological stages of sporulation in ERIC I (strain-7841) and ERIC II (strain-7853) grown on solid MYPGP supplemented with 20% glucose. Images show the transition from vegetative cells through asymmetric septation, engulfment, and forespore maturation, to phase-bright spore formation: scale bar, 2 µm. Statistical analysis of the sporulation efficiency of strains in both solid and liquid media in response to specific sugars was performed using two-way ANOVA followed by Tukey’s multiple comparison test. Significance levels are indicated as follows: p**** < 0.0001; ns, not significant.

Spore formation by *P. larvae* constitutes an important aspect of American foulbrood disease transmission. Thus, we wanted to explore if ERIC I and ERIC II strains exhibit differences in their ability to produce spores. To comprehensively assess the sporulation efficiency of ERIC I and ERIC II strains, we examined sporulation efficiency in both liquid broth and solid MYPGP media, supplemented with Glucose, Fructose, and Trehalose. In both genotypes, spore formation was consistently higher on solid media than in liquid media, regardless of the type of sugar used. Interestingly, on solid media, increasing the sugar concentration from 10% to 20% resulted in an approximately two-fold increase in sporulation efficiency, reaching around 7.0-8.5% in both ERIC I and ERIC II strains across all sugars tested (Figs 3A, 3B, S4A and S4B). However, in liquid media, regardless of the ERIC type, specific strain used or type of sugar supplemented, the maximum sporulation efficiency observed was only 0.5-1.5% (Figs 3A, 3B, S4A and S4B). Given the uniformly poor sporulation observed in liquid culture, we examined whether sugar type or ERIC genotype influenced sporulation under solid culture conditions. Previous studies have reported that ERIC I strains sporulate more efficiently in fructose, whereas ERIC II strains show higher sporulation in trehalose [20]. In contrast, under the conditions tested in the present study, no genotype-specific preference for fructose or trehalose was observed. Furthermore, although not statistically significant, both ERIC I and ERIC II strains produced comparable or slightly higher numbers of spores on solid media supplemented with glucose compared to fructose and trehalose (S4C Fig).

To further assess whether ERIC I and ERIC II strains differ in sporulation, we monitored sporulation progression microscopically in ERIC I strain 7841 and ERIC II strain 7853 by collecting culture samples daily, from solid MYPGP medium supplemented with 20% glucose over a period of 8 days (Fig 3C). By day 3, in both the ERIC I and ERIC II strains selected, we observed cells that had entered sporulation as evidenced by the characteristic polar septum and engulfing membranes which mark early stages of sporulation [21]. By day 4, we observed the formation of phase-bright spores in the ERIC I strain but not the ERIC II strain, which only produced phase-bright spores by day 6 (Fig 3C). Notably, by day 8, many phase-bright spores in both ERIC I and ERIC II strains were no longer contained within the mother cells, indicating mother cell lysis. Although the selected ERIC II strain appeared slower to develop phase-bright spores than the ERIC I strain, both strains progressed through the different stages of sporulation at a comparable rate.

### Structural characterisation of mature spores in ERIC I and ERIC II strains

**Fig 4:**
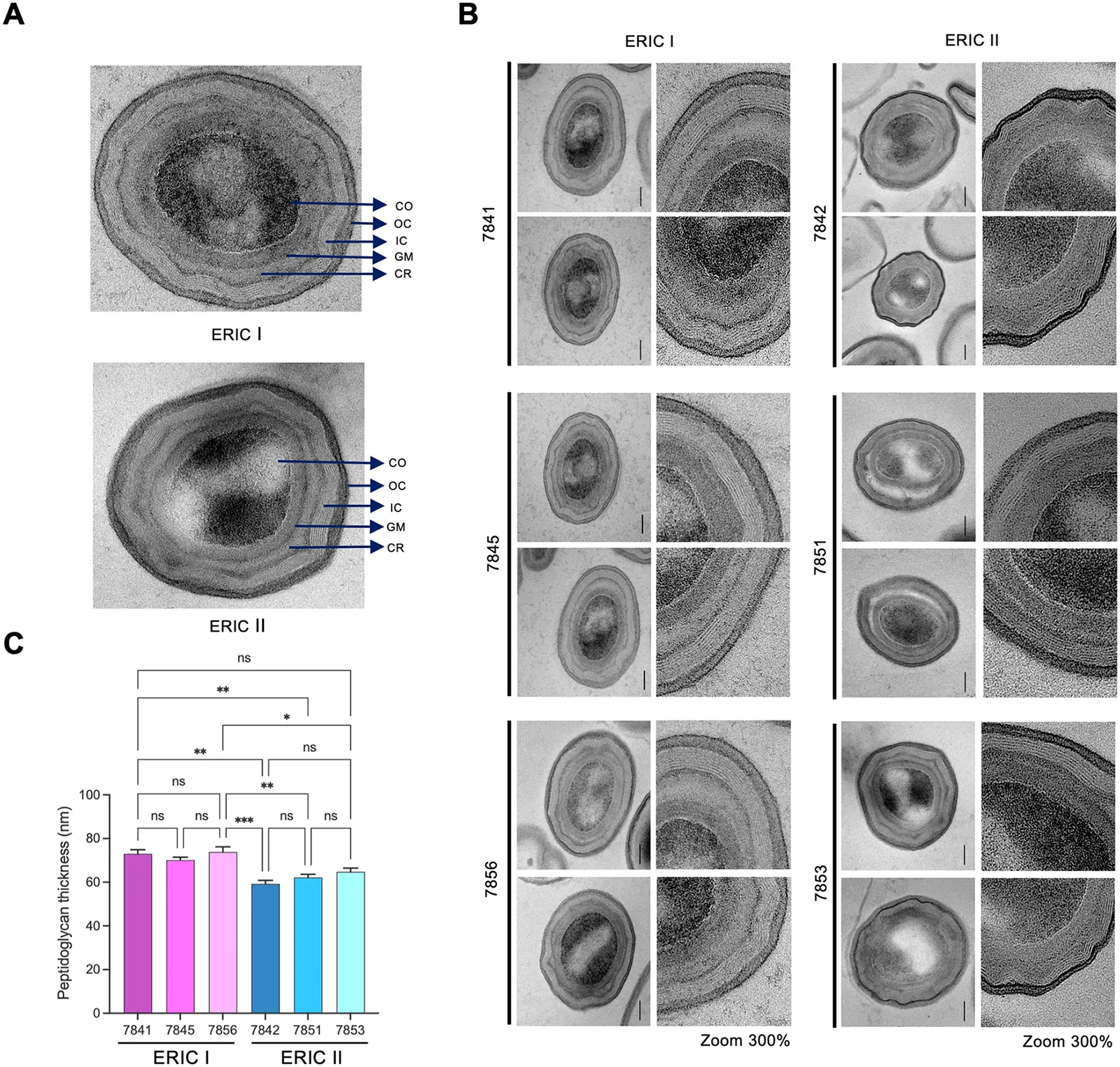
Comparison of ERIC I and ERIC II endospores by Transmission Electron Microscopy. **(A)** Representative micrograph of *P. larvae* ERIC I (strain-7841, 7845, 7856) and ERIC II (strain-7842, 7851, 7853) endospores by TEM, indicating the different spore envelope layers. Abbreviations: CO, spore core; OC, outer spore coat; IC, inner spore coat; GM, germ cell wall; CR, cortex. **(B)** Representative micrograph of spores from the three ERIC I and three ERIC II strains. Scale bar, 100 nm. For each strain, the left panel shows the entire endospore, and the right panel presents a higher-magnification (300% zoom) view of ultrathin cross-sections **(C)** Peptidoglycan thickness in ERIC I and ERIC II strains. Data are presented as (mean ± SD) of three independent biological replicates. Significant differences in peptidoglycan thickness among the strains were determined using one-way ANOVA followed by Tukey’s multiple comparison test. Significance levels are indicated as follows: p* < 0.05, p** < 0.01, p*** < 0.001; ns, not significant.

Transmission electron microscopy (TEM) has classically been used to examine the structure of bacterial spores, allowing the observation of the various spore envelope layers [22]. The spore envelope of *P. larvae* has yet to be carefully examined using this approach, and it remains unclear if the structure of ERIC I and ERIC II spores differs or not. We therefore examined spore envelope structure in three ERIC I and three ERIC II strains using TEM, based on the established spore architecture of endospore-forming bacteria such as *Bacillus subtilis* and *Paenibacillus polymyxa* [23,24]. We could clearly identify the distinct cellular layers of the spore, including the spore core, germ cell wall, cortex and coat in the ERIC I and ERIC II strains (Figs 4A, 4B and S5). The spores of the ERIC I and ERIC II strains were comparable, and we could not identify major differences between ERIC types or between strains except for the thickness of their peptidoglycan layer (germ cell wall and cortex combined): the peptidoglycan layer in ERIC I occupies more space in the spore envelope than that observed for the ERIC II strain (Fig 4C). Interestingly, compared to well-documented TEM images of *B. subtilis*, a well-studied, model spore forming bacterium, we observed a thicker and visibly distinct germ cell wall in ERIC I and ERIC II strains of *P. larvae* selected for this study. The germ cell wall, at least in *B. subtilis,* is typically thought of as a thin layer of peptidoglycan and is often not easy to observe by TEM [23]. The thickness and ease of observation of the germ cell wall of *P. larvae* spores by TEM indicate that this peptidoglycan layer could be thicker in this organism. Collectively, these data suggest that the spores produced by ERIC I and ERIC II strains have similar structural architecture and that *P. larvae* produce spores with a thicker germ cell wall, compared to well-studied *B. subtilis*.

### Germination dynamics of ERIC I and ERIC II strains

**Fig 5:**
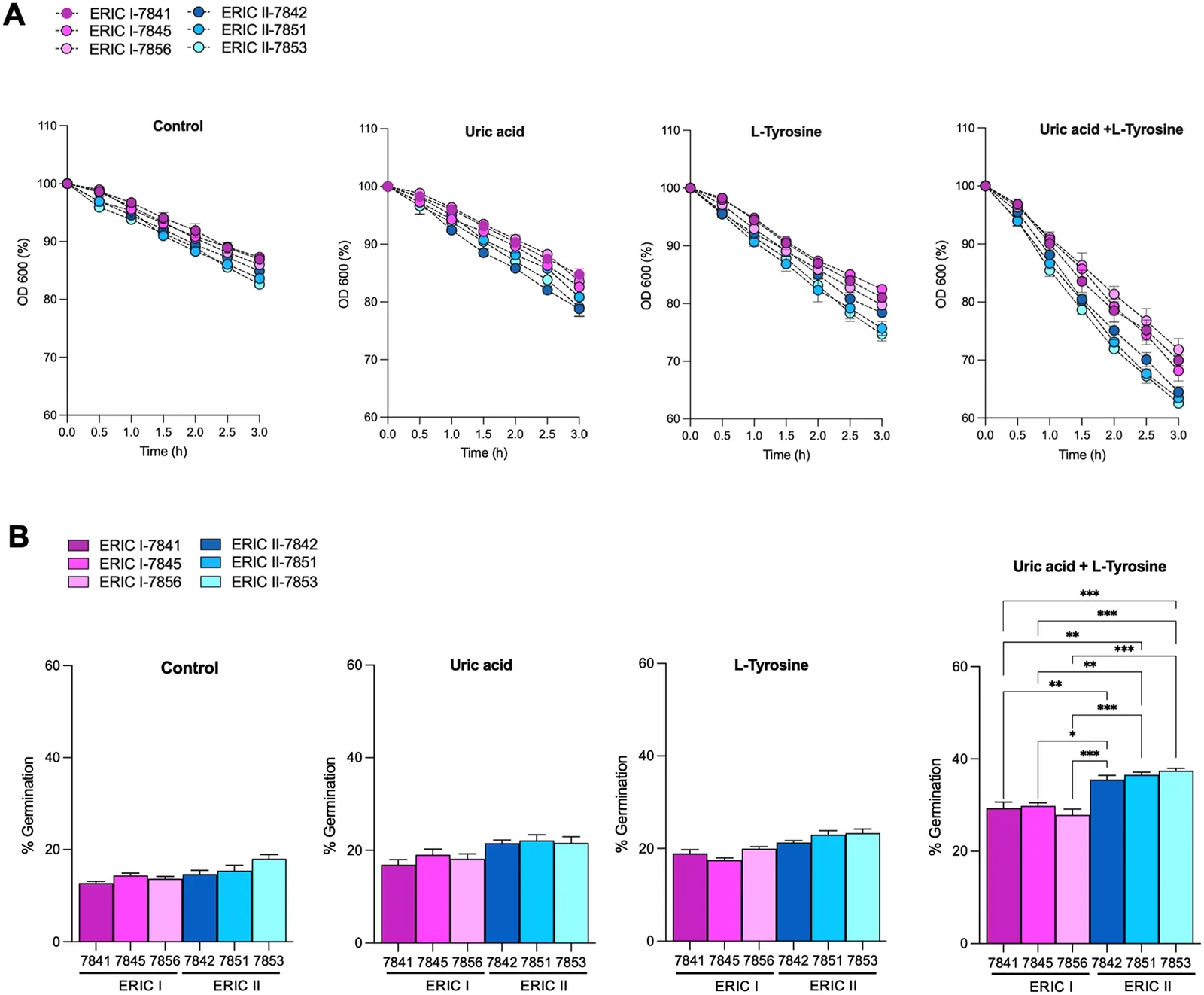
Spore germination efficiency and germination rate of ERIC I and ERIC II strains. **(A)** Spore germination dynamics of ERIC I and ERIC II strains in response to different germinants: Uric acid, L-Tyrosine, a combination of Uric acid and L-Tyrosine, and without any germinants (control). Spore germination is indicated by the percentage reduction in OD₆₀₀ over time. Data are presented as (mean ± SD) of three independent biological replicates. **(B)** Germination rate of ERIC I and ERIC II strains under different conditions as described in **(A),** over the first three hours. Values are expressed as (mean ± SD) of three independent biological replicates. Columns labelled with different asterisks indicate statistically significant differences in germination rate among the strains, determined using one-way ANOVA followed by Tukey’s multiple comparison test. Significance levels are indicated as follows: p* < 0.05, p** < 0.01, p*** < 0.001; ns, not significant.

A critical stage of the infection process by *P. larvae* is the germination of their spores in newly hatched bee larvae. As mentioned in the Introduction, ERIC I and ERIC II exhibit differences in the rate at which they cause infection and death of bee larvae. Therefore, we considered the possibility that ERIC I and ERIC II strains may have different germination dynamics, which could influence the speed at which these strain types initiate gut colonisation, thereby affecting the progression of infection and bee larval mortality. To test this hypothesis, we examined the germination dynamics of ERIC I and ERIC II strains using an *in vitro* broth-based germination assay. Purified spores were incubated for 18 h in MYPGP medium alone, or in MYPGP medium supplemented with the known germinants: uric acid, L-tyrosine or a combination of both (S6 Fig) [25]. Across all six *P. larvae* strains examined, the addition of germinants markedly increased spore germination compared with the no-germinant (control), although the efficiency of germination varied among strains and between genotypes (Fig 5A). As expected, the germination efficiency was highest in media supplemented with both uric acid and L-tyrosine (27–38%), whereas lower germination efficiencies were observed in media containing only one germinant (17–22%) or no germinants (15–18%) (Fig 5B). However, ERIC II strains consistently showed higher germination efficiencies than ERIC I strains in the presence of germinants (Fig 5A), and this difference was further evident by statistical analysis of the germination rates (Fig 5B).

## Discussion

Critical to a better understanding of *P. larvae* infection biology is a detailed study of the genetics and cellular biology of the pathogen itself. In this study, we sought to identify genetic and morphological differences in the growth, sporulation and germination of the two major strain types of *P. larvae*, ERIC I and ERIC II. Consistent with previous reports [26,27], our comparative genomic analysis of the *P. larvae* strains revealed clear distinctions between ERIC I and ERIC II groups, alongside substantial conservation of core genetic content (Figs 1A, 1B and S1A). Analyses of their pan-genomes corroborated these genomic trends. Most genes were shared, emphasising vertical inheritance of the core genome, while accessory genes contributed to ERIC-specific differences. The long branches separating the two ERIC groups, together with the relatively short genetic distances observed among strains within each group, are consistent with an early divergence of these lineages in the evolutionary history of the species, as previously suggested [28], followed by relative genetic stability within each clade. Furthermore, strains originating from different countries were distributed across both ERIC groups and formed coherent clusters. This pattern likely reflects extensive beekeeping practices, including the movement of bees, hive products, and beekeeping equipment, which have facilitated the widespread dissemination of dominant *P. larvae* types across different regions [29]. Importantly, the wide phylogenetic distribution of the strains analysed here suggests that the phenotypic differences identified in this study are not caused by a small number of closely related isolates, but instead reflect broader features associated with ERIC I and ERIC II genotypes.

The clear genomic divergence between ERIC I and ERIC II strains suggests that their growth patterns and nutrient preferences may be influenced by specific genetic features. Under controlled laboratory conditions, growth assays showed that both ERIC I and ERIC II strains grew similarly when glucose was provided as the main carbon source, with comparable generation times (S2 Fig). This indicates the core metabolic processes supporting growth on glucose are largely conserved between the two ERIC types, despite their genomic differences. Differences in growth became evident when alternative sugars were tested. ERIC I strains consistently demonstrated faster growth on fructose, whereas ERIC II strains exhibited shorter generation times on trehalose (Figs 2A, 2B and S2). These contrasting growth patterns indicate differences in carbohydrate utilisation, likely determined at the genomic level. Such variation may also arise from differences in sugar transport systems, regulatory networks, or downstream metabolic pathways, and identifying the specific genetic determinants will require further investigation. Trehalose is the dominant circulating sugar in insects and plays a central role in insect energy metabolism [30]. The faster growth of ERIC II strains on trehalose may contribute to their fitness during infection, particularly when bacterial cells encounter host-derived nutrients [31]. In contrast, the ability of ERIC I strains to utilise fructose may enhance their competitiveness in hive-associated environments, where fructose is abundant in nectar-derived substrates [32]. From an applied beekeeping perspective, these findings suggest that optimising sugar composition in supplemental larval feeding may help limit bacterial proliferation and minimise associated disease risk. While laboratory conditions cannot fully replicate the complexity of natural environments, the observed metabolic differences require *in vivo* validation. However, these findings provide a potential physiological basis for understanding previously observed variation in disease progression and virulence among ERIC genotypes [33,34].

Our morphological differences between *P. larvae* ERIC I and ERIC II strains are conserved across isolates and growth phases. ERIC I strains consistently produced longer cells than ERIC II, and quantitative image-based measurements confirmed this distinction, with ERIC I cells being, on average, at least fourfold longer than ERIC II cells during log phase (Fig 2D). These findings extend earlier observations with single representative strains [18] and establish that cell length is a stable, ERIC-associated phenotypic trait of *P. larvae*. The reason for the differences in length between ERIC I and ERIC II strains is not clear at present, but several possibilities exist. In rod-shaped bacteria, cell length is primarily controlled by the balance between elongation and division, which is guided by cytoskeletal proteins such as FtsZ [35]. Genetic differences between ERIC types, which are known to influence virulence, toxin production, and disease progression [36,37], may also affect genes that control the cell cycle or peptidoglycan synthesis. The longer cells observed in ERIC I strains could arise from delayed division or extended elongation during rapid growth, potentially connecting cell shape to physiological or pathogenic traits. However, both ERIC I and ERIC II strains became shorter during the stationary phase, reflecting the typical bacterial response to nutrient limitation and indicating conserved regulation of cell wall synthesis and division at least during this stage of growth [38–40]. Supporting this interpretation, comparative analysis of the division and cell wall (DCW) gene cluster revealed a high degree of conservation between ERIC I and ERIC II strains (S1C Fig), suggesting that the core machinery controlling cell elongation and division is preserved. Thus, ERIC-specific morphological differences are unlikely to arise from large-scale structural variation and are more likely linked to subtle regulatory effects, differences in gene expression, or interactions with upstream pathways that control growth [41,42]. An important methodological insight from this study is that plasmid-based genetic manipulation significantly alters *P. larvae* cellular morphology. Introducing a GFP-expressing plasmid increased cell length in both ERIC backgrounds (S3B and S3D Fig). Similar effects of plasmid maintenance and expression of foreign fluorescent proteins have been reported in other bacterial systems [43], highlighting the sensitivity of cell division processes and showing that foreign proteins can stress the cell and interfere with division [44].

In our study, we found that sporulation in *P. larvae* was strongly influenced by growth conditions, with both ERIC I and ERIC II strains producing substantially more spores on solid media than in liquid culture (Fig 3A and 3B). Surface-associated growth is known to promote sporulation in many Gram-positive spore formers, as it creates localised nutrient limitations, cell-density effects, and microenvironmental heterogeneity that favour entry into the sporulation pathway [45–47]. In contrast, liquid cultures provide more homogeneous nutrient availability, which can delay or suppress sporulation initiation, consistent with the uniformly low sporulation efficiencies reported in [48]. The increased sporulation observed at higher sugar concentrations on solid media may be due to enhanced vegetative growth before sporulation, allowing cells to accumulate sufficient energy and metabolic reserves to complete the energetically demanding sporulation process (Fig 3A and 3B) [49,50]. Under these conditions, sugar concentration appeared to have a stronger influence on sporulation efficiency than sugar type or ERIC genotype.

Transmission electron microscopy revealed that ERIC I and ERIC II spores share a conserved structural organisation, but differ in peptidoglycan thickness, with ERIC I spores possessing a thicker peptidoglycan layer overall (germ cell wall and cortex combined). The peptidoglycan layer is a rigid structural component that surrounds the bacterial membrane, provides mechanical strength, maintains cell shape, and protects against osmotic stress and lysis [51].

In endospores, the modified cortex peptidoglycan contributes to core dehydration and the maintenance of dormancy, and its controlled hydrolysis is essential for initiating germination; on the other hand, the germ cell wall facilitates uniform deposition of the cortex and acts as the peptidoglycan of the germinating spore [52]. An overall thicker peptidoglycan layer in ERIC I spores may contribute to increased resistance to adverse conditions, as spore peptidoglycan and cortex structure are known to play important roles in spore resistance properties [51]. Conversely, a thinner peptidoglycan layer, as observed in ERIC II spores, may facilitate more rapid degradation during germination, potentially leading to more efficient germination [53]. In addition to differences in overall peptidoglycan thickness, *P. larvae* spores displayed a thicker germ cell wall compared to the model organism *Bacillus subtilis,* reflecting species-specific adaptations that enhance spore stability and regulate germination. The thicker germ wall could indicate the need to protect the outgrowing spore, as it transitions into the vegetative cell wall upon germination in the larval gut environment [54,55].

Spore germination is a key step in the infection process of *P. larvae*, as only germinated spores can proliferate and colonise the gut of newly hatched honeybee larvae [56]. Germinants are thought to act by binding to specific germinant receptors located in the spore inner membrane, triggering a cascade of events that lead to cortex degradation, water uptake, and resumption of metabolic activity [57]. In the present study, the addition of known germinants strongly enhanced spore germination compared with control conditions, supporting existing data that *P. larvae* spores require specific environmental signals to exit dormancy. The very low germination observed in the absence of germinants represents the highly resistant and dormant nature of the spores. The higher germination efficiencies observed in the presence of uric acid and L-tyrosine are consistent with previous work identifying these compounds as effective germinants for *P. larvae* spores [25]. The enhanced response to the combined germinants suggests that spores integrate multiple signals to trigger germination, which may provide more favourable conditions inside the gut of the bee larvae. Importantly, ERIC II strains consistently showed slightly higher germination efficiencies and faster germination rates than ERIC I strains under germinant-supplemented conditions (Fig 5A and 5B). This slight difference may contribute to the faster onset of infection and more rapid larval death associated with ERIC II strains [58,59]. Furthermore, these findings suggest that strain-specific differences in spore germination could be an important factor influencing the progression of *P. larvae* infection in honeybee larvae.

### Limitations of the study

While this study advances our understanding of *P. larvae* biology, a key limitation is that the findings may not fully reflect the biology of this pathogen during *in vivo* infection. Laboratory-cultivated organisms often exhibit different behaviours compared with those grown within the host or under host-mimicking conditions. Therefore, it is essential to examine and compare *P. larvae* growth, sporulation, and germination under both *in vitro* and *in vivo* conditions to identify characteristics specific to each of these conditions.

### Conclusion

In this study, we provide a comprehensive analysis of the genetic and phenotypic differences between ERIC I and ERIC II strains of *Paenibacillus larvae*. Our results show that the two ERIC types differ in growth, cellular morphology, spore envelope architecture, and genomic features. ERIC I strains have larger genomes and harbour a larger number of prophage-derived regions and a broader repertoire of CRISPR-Cas and resistance- or competition-associated loci, along with longer vegetative cells and thicker spore walls, all of which likely enhance their survival and competitive ability in the hive. In contrast, ERIC II strains germinate faster, their genomes encode a more streamlined accessory gene set but frequently contain a lineage-specific integrative and conjugative element (ICE) together with efflux-related resistance determinants, reflecting a different strategy for environmental adaptation and horizontal gene transfer that may promote rapid infection of honeybee larvae. These differences reflect stable, ERIC-associated traits rather than isolate-specific variability and suggest that each ERIC type has evolved strategies suited to distinct ecological niches and infection dynamics. Overall, our findings improve understanding of *P. larvae* biology and provide a foundation for developing more targeted, genotype-specific disease management strategies for American foulbrood (AFB).

## Materials and methods

### Selection of strains

*Paenibacillus larvae* strains were selected based on their geographic origin and the epidemiological significance of American foulbrood (AFB). The genotypes of the selected strains were previously characterised using the multilocus sequence typing (MLST) scheme described in Morrissey *et al*. [60]. The isolates were obtained from the UK Food and Environment Research Agency (FERA), York, UK, and are listed in Table 1. For phenotypic characterisation, only the six *P. larvae* isolates were used to assess strain-specific variability within each ERIC type, and no additional reference or control strains were included.

### Culture of *Paenibacillus larvae*

To establish optimal growth conditions of *P. larvae,* we slightly modified an existing protocol [61] to support growth in both liquid and solid media. Briefly, *P. larvae* strains were streaked onto MYPGP agar plates containing 12% glucose and supplemented with nalidixic acid (20 µg/mL) and pipemidic acid (10 µg/mL). The incorporation of Nalidixic acid in the culture medium inhibited the growth of *P. alvei* and Pipemidic acid, preventing the development of most other *Bacillus* and *Paenibacillus* species, which normally develop on plates before *P. larvae* [62]. Isolates recovered on MYPGP agar supplemented with nalidixic acid and pipemidic acid were subcultured once on non-selective BHI agar to eliminate potential sugar pre-adaptation effects. Following this intermediate passage, single colonies were selected and used to assess the effect of different saccharides on bacterial growth by culturing the strains overnight in MYPGP broth supplemented with either glucose, fructose, or trehalose, and adjusted to an OD₆₀₀ of 0.05. Bacterial growth in the respective sugar-supplemented MYPGP broths was subsequently monitored over a 24 h period using a Tecan microplate reader. Generation time was calculated from data obtained during the exponential growth phase. Growth curves were fitted to the exponential model *y = A*e^*B*x^, where *y* represents the optical density at time *x*, *A* is the initial optical density, and *B* is the growth rate constant. The generation time (*g*) was calculated using the equation *g = ln(2)/B*. Preparation of precultures for genetic manipulation was carried out by inoculating a freshly isolated single colony into 5 mL of MYPGP broth and incubating at 37 °C for 18 h with shaking at 200 rpm. This preculture was used to inoculate a new culture in the morning, consisting of 25 mL of fresh MYPGP at a starter OD₆₀₀ of 0.05, and incubated at 37 °C with shaking (200 rpm) until cells reached the early log-phase. For plasmid propagation, the GFP expression vector pAD43–25 was introduced into *Escherichia coli* DH5α cells by a standard heat-shock. Transformed cells were selected and grown on Luria-Bertani (LB) agar and in LB broth supplemented with ampicillin (100 µg/mL). Genetic manipulation of the selected *P. larvae* strains was conducted with slight modification of the protocol described in Poppinga et al. [18]. Briefly, different chloramphenicol concentrations were evaluated for strain selection, and 30 µg/mL was determined to provide optimal selection efficiency for the strains used in this study, compared with the 5 µg/mL concentration described in the original protocol. Recombinant *P. larvae* strains were subsequently selected on MYPGP agar plates containing chloramphenicol (30 µg/mL) and incubated at 37 °C for 3–4 days.

### Whole genome sequencing and genome analysis

*Paenibacillus larvae* strains were streaked and cultured on MYPGP agar plates at 37°C for 72 hours. Single colonies were selected and grown overnight in MYPGP broth. Bacterial cells were harvested by centrifugation at 6,000 x g, and genomic DNA was extracted from the bacterial pellet using the DNeasy Blood & Tissue Kit (Qiagen, Hilden, Germany) according to the manufacturer’s instructions. The quality and quantity of the extracted DNA were assessed using a NanoDrop 2000 spectrophotometer (Thermo Fisher Scientific, Waltham, USA). Whole genome sequencing was performed on an Illumina NovaSeq 6000 platform [63] Novogene, UK) to achieve an average coverage of 100X. The quality of raw reads was assessed using FastQC (v0.11.9) [64]. Adapter sequences and low-quality ends of reads were trimmed by Trimmomatic (v0.39) [65]. Filtered reads were then assembled de novo using SPAdes (v3.14.0) [66]. The assembled genomes were consequently annotated using the Prokka annotation pipeline (v1.13) [67] and screened for AMR-associated genes against CARD database (entries from April 2023) [68] using ABRicate. PhiSpy (v4.2.21) [69] was used to aid prophage region identification. Genome completeness and quality were assessed with QUAST (v5.0.2) [70]. The draft genomes were mapped, reordered, and compared against two NCBI reference genomes of *P. larvae* (ATCC 9545-accession number NZ_CP019687.1 and DSM25430-accession number NZ_CP003355.1) using BRIG (v0.95) [71]. ERIC-specific genes initially annotated as hypothetical proteins were further analysed against the UniProtKB/Swiss-Prot database in an effort to assign putative functional annotations [72]. Pan-genome analysis of those eight strains was performed employing Roary (v3.13) [73], core-genomes were aligned using MAFFT (v7.525) [74]. A phylogenetic tree based on the core genome of eight strains was inferred using IQ-Tree (v2.4) [75] with the GTR + F + R4 substitution model and faster tree-search heuristics. Orthologous protein clustering was performed using OrthoVenn3 [76], and gene clustering was analysed using Clinker [77].

### Phylogenetic and co-conservation analysis

For phylogenetic and co-conservation analyses, we used six newly sequenced *P. larvae* isolates obtained from different geographical locations, representing a balanced subset of ERIC I and ERIC II genotypes (three isolates per ERIC type), together with 782 publicly available *P. larvae* complete genomes retrieved from the NCBI database in September 2025. Multilocus sequence typing (MLST) profiles were identified *in silico* using the *P. larvae* MLST scheme described in the PubMLST [78]. Allelic profiles were assigned through BLAST-based comparison, and sequence types (STs) were cross-validated against existing entries in the database. Assemblies were annotated using Bakta (v6.0) [79] and subsequently processed with Roary [73] to generate a core-genome alignment. The alignment was filtered to remove paralogous loci and poorly aligned regions. Phylogeny reconstruction was conducted using IQ-Tree (v2.4) [75] under a maximum likelihood framework with 1,000 ultrafast bootstrap replicates to assess branch support. Pairwise patristic distances were calculated from the maximum-likelihood phylogeny using a custom Python script implemented using Biopython (v1.82) [80]. The best-fit substitution model was selected automatically using ModelFinder integrated within IQ-TREE. To evaluate the influence of homologous recombination on the inferred phylogeny, the ML tree and corresponding core-genome alignment were analysed using ClonalFrameML [81]. This program estimates the relative effect of recombination to mutation (r/m) and corrects branch lengths according to the detected recombination events. Phylogenetic trees were visualised and annotated in iTOL (v6) [82]. The combination of whole-genome phylogenetic analyses with MLST and ERIC typing data enabled a direct comparison between traditional genotyping approaches and genome-scale evolutionary relationships.

### Sporulation assays

To develop an optimised laboratory protocol for *Paenibacillus larvae* spore production, we slightly modified a previously described method [61] and prepared MYPGP agar plates and broth supplemented with different sugars, including glucose, fructose, and trehalose, to support growth in both solid and liquid media. Briefly, *P. larvae* strains were initially streaked onto MYPGP agar plates to obtain individual colonies. For spore production in liquid medium, single colonies were inoculated into 15 mL of MYPGP broth containing the respective sugars and incubated at 36 °C with shaking at 200 rpm for 10 days. For spore production on solid medium, overnight cultures of *P. larvae* strains were prepared, and 200 µL of culture was adjusted to an OD₆₀₀ of 0.1 and then spread onto MYPGP agar plates containing individual saccharides. Plates were incubated at 36°C for 10 days. Bacterial lawns were harvested by rinsing the plate surfaces three times with 1 mL of sterile, cold (4°C) deionised water and gently scraping the colonies using a cell spreader. Cells grown in liquid medium were collected by centrifugation (3000 × g, 10 min, 4 °C). Cells harvested from both solid and liquid media were processed separately. Scraped colonies from solid media were first washed three times with 10 mL PBS, followed by four washes with 15 mL sterile, cold deionised water by repeated vortexing and centrifugation (3000 × g, 15 min, 4 °C) in 15 mL of sterile, cold deionised water using 50 mL centrifuge tubes. After discarding the supernatant, pellets were resuspended in cold deionised water to prepare spore stocks. The spore suspensions were heat-treated at 65 °C for 15 min to eliminate vegetative cells and heat-sensitive immature spores. Following serial decimal dilutions, spore suspensions were plated on MYPGP agar supplemented with germination factors (L-tyrosine and uric acid) and incubated at 35 °C for 5 days in biological triplicate. The number of viable, heat-resistant spores was determined as colony-forming units (cfu/mL) and sporulation efficiency was calculated using the following formula**:** (Number of heat-resistant spores/ Number of total viable cells) × 100**.**

### Chemical fixation, thin sectioning, and TEM of *P. larvae* spores

Spore pellets were fixed in 3% (w/v) glutaraldehyde in 0.1 M sodium cacodylate buffer at 4°C overnight. Samples were washed three times with fresh cacodylate buffer to remove excess glutaraldehyde. Secondary fixation was performed with 1% (w/v) osmium tetroxide for 2 hours, followed by three further washes with Milli-Q water. Spore pellets were dehydrated by incubating with increasing concentrations of ethanol (50%, 75%, 95%, 99%, 100%) for 10 minutes. Dehydrated samples were further incubated twice with propylene oxide for 15 minutes. Overnight infiltration occurred with a 1:1 mix of propylene oxide and Araldite resin; the resin was removed, and excess propylene oxide allowed to evaporate at room temperature. Samples were incubated with fresh araldite resin for two consecutive four-hour periods, before final embedding in fresh resin. Resin was polymerised at 60°C for 72 hours. Sections (70nm) were produced using a Powertome PC Ultramicrotome (RMC Boeckeler) and floated onto 300-square mesh nickel TEM grids. Sections were post-stained in 3% (w/v) uranyl acetate for 30 minutes, washed three times with Milli-Q water, stained with 1% lead citrate for 5 minutes and washed a further three times with Milli-Q water. Sections were imaged on a Jeol2100Plus 200kV microscope, at a magnification of 40k (0.275 nm/pixel), equipped with a Gatan OneView IS camera.

Peptidoglycan thickness in TEM images was measured as the distance between the spore core and coat, corresponding to the germ cell wall and cortex layers, as described in the canonical endospore architecture of Firmicutes [23,24]. Measurements were taken at five different positions within each spore image, and the mean value was calculated. Fifteen spores were analysed for each strain across three biological replicates, and the mean of these measurements was used to represent the peptidoglycan thickness for that strain. All measurements were obtained using the Scale Bar tool in Fiji (ImageJ) [83].

### Germination assays

Heat-killed spores were washed twice with double-distilled water (ddH_2_O) by centrifugation at 3000 × g for 10 min. The resulting spore pellet was resuspended in 350 µL of 20% Histodenz (Sigma-Aldrich) and carefully layered onto 1 mL of 50% Histodenz, followed by centrifugation at 3000 × g for 35 min. The pellet fraction was collected and washed five times with 1 mL of ice-cold ddH_2_O before final resuspension in 500 µL ddH_2_O. The presence and purity of spores were confirmed by phase-contrast microscopy. After that, purified spores were adjusted to an OD₆₀₀ of 1.5 in ddH_2_O. For germination assays, 250 µL of the spore suspension was added to each well of a clear 48-well plate (StarLab), followed by the addition of 250 µL of MYPGP broth. Germination assays were performed with the addition of a single germinant (either 3 mM uric acid or 3 mM L-tyrosine), a combination of both germinants (3 mM uric acid and 3 mM L-tyrosine) and without any germinants. The germination of *P. larvae* strains (ERIC I and ERIC II) was evaluated over a period of 18 hr, with time points measured at every half-hour interval, and data were normalised to 100% at 0 hours. The spore germination rate (%) was calculated as the total decrease in OD₆₀₀ relative to the initial measurement at the onset of the assay, using the following equation:


$$\begin{array}{c} \text{Germination }(\%)=\frac{{\text{OD}}_{\text{initial}}-{\text{OD}}_{\text{final}}}{{\text{OD}}_{\text{final}}}\times \text{100} \end{array}$$

### Fluorescence microscopy

A single colony was selected from a freshly streaked MYPGP agar plate, with antibiotic selection applied where required, and grown overnight in 5 mL of MYPGP broth at 25 °C with shaking at 200 rpm. The overnight culture was diluted into 20 mL of fresh MYPGP broth to an OD₆₀₀ of 0.05 and incubated at 37 °C with shaking at 200 rpm until cultures reached either the mid-logarithmic or stationary growth phase. From the respective growth phases, 300 µL of the culture was pelleted by centrifugation and resuspended in 10 µL of 0.05% TMA-DPH [1-(4-trimethylammoniumphenyl)-6-phenyl-1,3,5-hexatriene p-toluenesulfonate] membrane dye prepared in MYPGP medium. Subsequently, a 1.5-µL aliquot of the stained cell suspension was placed onto a 2% agarose pad prepared with MYPGP medium and sealed using a Gene Frame (Bio-Rad). Images were acquired using a Zeiss Observer 7 microscope equipped with a Plan-Apochromat 100 × /1.4 Oil Ph3 objective and a Colibri 7 Type R[G/Y] CBV-UV light source. TMA-DPH fluorescence (0.05 mM) was excited using a Zeiss 92HE filter set with an exposure time of 100 ms. GFP fluorescence was imaged using a Zeiss Axio 108HE filter set with exposure times of 1000 ms and 800 ms, respectively.

### Image analysis and statistics

Phase contrast images were analysed using the MicrobeJ plugin in Fiji [84] to measure cell length (µm) and width (µm). Automated segmentation was performed, and cell outlines were inspected and manually corrected using the corresponding TMA-DPH and/or GFP fluorescence signals. For each biological replicate, at least 100 cells were analysed. All statistical analyses were conducted using Python (v3.14.3) [85], and statistical significance was defined as *p* < 0.05. All the graphs were generated using GraphPad Prism (v8.2.1) [86].

## Supporting information

S1 Fig Phylogenetic, protein clustering analysis, and DCW cluster organisation. **(A)** Maximum likelihood (ML) phylogenetic tree illustrating the genetic relationships among *P. larvae* isolates. MLST sequence types and strains newly sequenced for this study are annotated. Branches are colour-coded according to their ERIC type, where sandy coloured clade is ERIC II and blue is ERIC I. **(B)** Orthologous protein clustering across genomes of the six novel strains from this study. The bar plot (top) shows the number of proteins in each orthologous cluster. The matrix plot (bottom) displays the presence and distribution of orthologous protein clusters across individual genomes, where each coloured point represents a protein cluster present in a given sample. **(C)** Genetic organisation of the division and cell wall (DCW) gene cluster in ERIC I and ERIC II strains. Genes are represented as different coloured, arrows indicating their relative positions and transcriptional orientations within the cluster. Connecting lines indicate sequence similarity. Scale bars represent genomic length in kilobases (kb).
(TIFF)

S2 Fig Growth curves of ERIC I and ERIC II strains. Representative growth curves of ERIC I and ERIC II strains over a period of 24h in MYPGP supplemented with glucose (Aqua), fructose (clover) and trehalose (plum). The data are presented as (mean ± SD) of three independent biological replicates.
(TIFF)

S3 Fig Cell morphology of ERIC I and ERIC II strains containing GFP encoded on a replicative plasmid. **(A)** Representative micrographs of ERIC I and ERIC II strains in the log-phase grown in MYPGP media. Scale bar, 2 µm. **(B)** Superplots displaying measurements of single-cell length and width for ERIC I and ERIC II strains in the log-phase. **(C)** Representative micrographs of ERIC I and ERIC II strains in the stationary phase. Scale bar, 2 µm. (**D**) Superplots displaying measurements of single-cell length and width for ERIC I and ERIC II strains in the stationary phase. **(E)** Superplots comparing cell length between non-transformed (WT) and transformed (plasmid-carrying) strains within the same genetic background ERIC I and ERIC II during the mid-log growth phase and **(F)** during the stationary phase. The data presented in superplots were obtained from three independent biological replicates, with 100 cells per replicate. Individual data points represent single cells, and colours denote different replicates. Statistical significance of differences in length and width among strains within each ERIC group was assessed using the Kruskal-Wallis test followed by Dunn’s multiple comparisons test. Differences within the same ERIC group and between ERIC groups were assessed using the Mann-Whitney U test. Statistical significance is indicated as follows: p* < 0.05, p** < 0.01, p*** < 0.001, p**** < 0.0001; ns, not significant.
(TIFF)

S4 Fig Sporulation efficiency of ERIC I and ERIC II strains. Sporulation efficiency of **(A)** ERIC I and **(B)** ERIC II strains grown with 10% glucose, fructose or trehalose in liquid and solid MYPGP media. Data are presented as (mean ± SD) of three independent biological replicates. **(C)** Sporulation efficiency of ERIC I and ERIC II strains on solid MYPGP media supplemented with 20% of glucose, fructose or trehalose. Data are presented as (mean ± SD) of three independent biological replicates. Statistical analysis of the sporulation efficiency of strains in solid and liquid media in response to specific sugars was performed using two-way ANOVA followed by Tukey’s multiple comparisons test. Significance levels are indicated as follows: p* < 0.05, p** < 0.01, p*** < 0.001, p**** < 0.0001; ns, not significant.
(TIFF)

S5 Fig TEM images of ERIC I and ERIC II endospores. Additional representative TEM micrographs showing spore envelope layers in ERIC I (top) and ERIC II (bottom) strains. For each strain, the left panel shows the entire endospore, and the right panel presents a higher-magnification (300% zoom) view of ultrathin cross-sections. Scale bar = 100 nm.
(TIFF)

S6 Fig Spore germination and outgrowth efficiency of ERIC I and ERIC II strains. Spore germination efficiency of ERIC I and ERIC II strains in response to the addition of different germinants: Uric acid, L-Tyrosine, a combination of Uric acid and L-Tyrosine, and without any germinants (control). The maximum germination was set to 100%, and spore germination was followed by the percentage reduction in OD₆₀₀ over time. Data are shown as (mean ± SD) of three independent biological replicates for a period of 18 hours.
(TIFF)

S1 Table Summary of protein clustering across *P. larvae* ERIC I and ERIC II strains. (DOCX)
