# Supplementary material for: Comparative genomics and phenotypic divergence of ERIC I and ERIC II genotypes of Paenibacillus larvae, the causative agent of American Foulbrood disease
Source: PLoS Pathog. 2026 Aug 13;22(8):e1014507. doi: 10.1371/journal.ppat.1014507 (PMC13497268; doi:10.1371/journal.ppat.1014507)
Supplement: Supplementary file 1 [file ppat.1014507.s007.docx]

**Table S1: Summary of protein clustering across *P. larvae* ERIC I and ERIC II strains**

| Strain | Protein | Clusters | Singletons |
| --- | --- | --- | --- |
| ERIC I-7841 | 4964 | 4861 | 72 |
| ERIC I-7845 | 4933 | 4754 | 144 |
| ERIC I-7856 | 5012 | 4913 | 56 |
| ERIC II-7842 | 4317 | 4226 | 74 |
| ERIC II-7851 | 4439 | 4332 | 82 |
| ERIC II-7853 | 4329 | 4285 | 26 |
