# Supplementary material for: Comparative genomics and phenotypic divergence of ERIC I and ERIC II genotypes of Paenibacillus larvae, the causative agent of American Foulbrood disease
Source: PLoS Pathog. 2026 Aug 13;22(8):e1014507. doi: 10.1371/journal.ppat.1014507 (PMC13497268; doi:10.1371/journal.ppat.1014507)
Supplement: Supplementary file 2 [file ppat.1014507.s008.docx]

**Part I - Summary**
Please use this section to discuss strengths/weaknesses of study, novelty/significance, general execution and scholarship.

Presented for the peer-review paper titled “Comparative genomics and phenotypic divergence of ERIC I and ERIC II genotypes of Paenibacillus larvae, the causative agent of American foulbrood disease” submitted to Plos Pathogens provides novel information on genetic and phenotypic differences between the two major genotypes of *P. larvae* - ERIC type I and ERIC type II. The manuscript provides a strong and reliable experimental design for future in-depth in silico-in vitro-in vivo studies in P. larvae and highlights the importance of research in bacterial pathogenesis, especially for the American Foulbrood.

This manuscript is a well-written, well-characterized study that uses WGS and phenotypic testing to significantly advance understanding of the inherited genetic differences in P. larvae and their role in subsequent AFB pathogenesis. This has great potential to evolve into a more comprehensive, larger study to uncover previously undescribed aspects of AFB pathogenesis and subsequently inform evidence-based countermeasures in the field. In contrast, a couple of aspects of this manuscript need improvement and/or clarification to ensure that the work presented was conducted properly. Additionally, there are several minor mistakes/ incorrect statements that need to be changed and supported with proper citation, as they might mislead readers. Lastly, the wording of some sentences is confusing and needs to be fixed for clarity and readability.

**Part II – Major Issues: Key Experiments Required for Acceptance**
Please use this section to detail the key new experiments or modifications of existing experiments that should be absolutely required to validate study conclusions.

Generally, there should be no more than 3 such required experiments or major modifications for a "Major Revision" recommendation. If more than 3 experiments are necessary to validate the study conclusions, then you are encouraged to recommend "Reject".

(Limit 20000 Characters)

No major issues have been identified.

**Part III – Minor Issues: Editorial and Data Presentation Modifications**
Please use this section for editorial suggestions as well as relatively minor modifications of existing data that would enhance clarity.

(Limit 20000 Characters)

See line-by-line comments below.

NCBI SRA project data – will be released by the time the paper is published?

L40 – Foulbrood is a one-word term as it is the name of a disease rather than a characteristic of brood.

L60 – I would say European honey bee since you are referring to the A. mellifera.

L68 – sentence needs to be rewritten to more clearly indicate that AFB is a disease and P. larvae is its causal agent.

L76-77 – Please provide a reference to why early age larval susceptibility makes disease prevention particularly challenging?

L77 – please indicate the sequence of AFB pathogenesis eg “ingestion-proliferation in the midgut-invasion through midgut epithelium – septiceamia”, as the sentence implies that proliferation in the midgut and hemolymph happens in parallel.

L90 – please include ERIC type V as well as per Beims et al.

L92-93 – Please elaborate on how lower virulence in ERIC I allows for the broader dissemination of spores?

L96 – If ERIC type II leads to quicker larval death, how would this accelerate the AFB outbreak? Do you mean the spread or the scale of affected larvae? Please clarify.

L112 – For the entire set of phenotypic experiments, did you use any controls apart from your 6 tested isolates? I am referring to the ATCC 9545 strain and/or other *P. larvae* strain that have been described previously in the research literature? If no, please clearly indicate it.

L170, 346 – resistance to what?

L171 – please indicate percent identity and coverage for the antibiotic-resistant genes; also, provide a comment on their functionality to put in the context.

L195 – please provide the total number of isolates analyzed.

L203 – could you please add a measure for the “not too closely related”-ness? Eg branch length?

L210-211 – were there any indication of differences in the metabolism genes? If so and they are relevant to this experiment, please add them in here.

L230 – I assume it was done in broth?

L240 – could you please provide an absolute measure too?

L252 – it is a comparison between transformed vs not transformed or in between transformed ERIC I and ERIC II?

L270 – broth sporulation efficiency

L292 – run-on sentence?

L300, 617 – up to date, there was no indication of works on *P. larvae* spore morphology and anatomy. Please explain how you decided/confirmed *P. larvae* spore anatomy and cite appropriate references.

L307 – since there is a great degree of variation in spore composition and anatomy, why didn't you choose any other *Paenibacillus spp*. spores for a reference? Please advise.

L317 – you start this section indicating that germination happens in the honey bee larvae, yet there is no indication that your experiment was conducted in vitro, e.g., in the broth rather than in honey bee larvae; please rewrite accordingly.

L329 – provide absolute value? Eg how much fold/times higher germination than in media without germinants?

L412-415 – looks like this finding is or might be limited by the number of genomes analyzed in the study.

L449 – please provide a reference or rewrite as this might mislead readers re: spore persistence.

L476 – please add a couple of sentences/a small paragraph for limitations of this study; in my opinion, this would allow readers to appreciate your findings and put them in the right context.

L507 – What exact saccharides did you use?

L511-512 – In between original recovery culture on MYPGP, with nalidixic and pipemidic acids, did you culture P. larvae on non-selective standard MYPGP or other media, e.g., CBA, to prevent media-induced changes in phenotype? Just to be clear: if you are culturing *P. larvae* on 12% saccharide media and then transferring it directly to broth with glucose/fructose or trehalose, wouldn't you expect increased sugar consumption (a potentiation of the growth effect), since the isolate has been pre-adapted on the saccharide MYPGP agar?

L514 – I assume that 24 growth was monitored in sugar-supplemented MYPGP broth; however, it is not clear, please indicate

L526-527 – If the “slight modification of the protocol” is not included elsewhere, please add it in the manuscript, as it is essential to your work.

L530 – I see results for the ICE, prophages, and antibiotic resistance genes in the “Results” section, yet I couldn't find anything in the M&M section. Please add information on your *in silico* analysis for the above-mentioned features if you haven`t done so.

L555 – Please indicate that you analyzed X number of *P. larvae* isolates in the first sentence.

L578 – your references don`t match to the text, please fix it.

L590 – In my personal experience, when harvesting *P. larvae* spores, sometimes it is hard to properly homogenize scraped colonies with vortexing only. Were there any other homogenization methods you used? If so, please indicate; if not, then you are a lucky one!

L635 – Were there any other methods employed to verify germination success, e.g., direct microscopy of aliquots, DPA (dipicolinic acid) test, etc.? if so, please indicate.

Figure 4A. Please indicate what is the stratified layer of the spore between OC and IC? (marked in red)
