# Supplementary material for: Comparative genomics and phenotypic divergence of ERIC I and ERIC II genotypes of Paenibacillus larvae, the causative agent of American Foulbrood disease
Source: PLoS Pathog. 2026 Aug 13;22(8):e1014507. doi: 10.1371/journal.ppat.1014507 (PMC13497268; doi:10.1371/journal.ppat.1014507)
Supplement: Supplementary file 3 [file ppat.1014507.s010.docx]

**Dear PLOS Pathogens Editor,**

Thanks for guiding our manuscript through the review process. We are glad to see that the reviewers appreciate the quality and value of our study. We address their points below.

**Additional Editor Comments:**

Your manuscript was reviewed by three experts. All felt the work was solid but that the writing was unclear in several places (see the extensive edits from reviewer 1). Please address these concerns.

**Part I - Summary**
Please use this section to discuss strengths/weaknesses of study, novelty/significance, general execution and scholarship.

***Reviewer #1****: Presented for the peer-review paper titled “Comparative genomics and phenotypic divergence of ERIC I and ERIC II genotypes of Paenibacillus larvae, the causative agent of American foulbrood disease” submitted to Plos Pathogens provides novel information on genetic and phenotypic differences between the two major genotypes of P. larvae - ERIC type I and ERIC type II. The manuscript provides a strong and reliable experimental design for future in-depth in silico-in vitro-in vivo studies in P. larvae and highlights the importance of research in bacterial pathogenesis, especially for the American Foulbrood. This manuscript is a well-written, well-characterized study that uses WGS and phenotypic testing to significantly advance understanding of the inherited genetic differences in P. larvae and their role in subsequent AFB pathogenesis. This has great potential to evolve into a more comprehensive, larger study to uncover previously undescribed aspects of AFB pathogenesis and subsequently inform evidence-based countermeasures in the field. In contrast, a couple of aspects of this manuscript need improvement and/or clarification to ensure that the work presented was conducted properly. Additionally, there are several minor mistakes/ incorrect statements that need to be changed and supported with proper citation, as they might mislead readers. Lastly, the wording of some sentences is confusing and needs to be fixed for clarity and readability.*

We thank the reviewer for their generous and positive feedback on our work. Their specific comments are answered below.

**Reviewer #1:** 
 ***NCBI SRA project data – will be released by the time the paper is published?***

**Author response:** SRA archives are already publicly available under BioProject PRJNA1434993.

***L40 – Foulbrood is a one-word term as it is the name of a disease rather than a characteristic of brood.***

**Author response:** It has been corrected in the manuscript as suggested. *”Foulbrood’*

***L60 – I would say European honeybee since you are referring to the A. mellifera.***

**Author response:** The sentence has been revised as follows:

**Page 3, Line 75:** *“The European honeybee (Apis mellifera) is an important pollinator species globally, aiding in the pollination of approximately 90-130 commercial crops that are vital to the human diet”.*

***L68 – sentence needs to be rewritten to more clearly indicate that AFB is a disease and
P. larvae is its causal agent****.*

**Author response:** The sentence has been revised as follows:

**Page 3, Line 82:** *“Among these stressors, infectious diseases pose a serious threat to colony survival, particularly American foulbrood (AFB), the most destructive brood disease of honeybees worldwide, caused by* Paenibacillus larvae*, a Gram-positive, spore-forming bacterium”.*

***L76-77 – Please provide a reference to why early age larval susceptibility makes disease prevention particularly challenging?***

**Author response:** A reference has been added to support the statement that early larval susceptibility contributes to the difficulty of preventing American foulbrood.

**Page 22, Reference 8:** *“Hansen H, Brødsgaard CJ. American foulbrood: a review of its biology, diagnosis and control. Bee world. 1999 Jan 1;80(1):5-23.”*

***L77 – please indicate the sequence of AFB pathogenesis eg “ingestion-proliferation in the midgut-invasion through midgut epithelium – septiceamia”, as the sentence implies that proliferation in the midgut and hemolymph happens in parallel.***

**Author response:** The sentence has been revised to clarify the sequential progression of American foulbrood pathogenesis as suggested.

**Page 3, Line 91-92:** *“Following germination of spores in the larval midgut*, P. larvae *vegetative cells then proliferate at this location and eventually breach the midgut epithelium, invading the haemocoel and causing septicaemia”.*

***L90 – please include ERIC type V as well as per Beims et al.***

**Author response:** The sentence has been revised by including ERIC type V as per Beims et al.

**Page 3, Line 104-106:** “P. larvae i*s known to be an extremely variable pathogen, and through ERIC (Enterobacterial Repetitive Intergenic Consensus) typing, different* P. larvae *genotypes have been identified, namely ERIC I, II, III, IV and V.”*

***L92-93 – Please elaborate on how lower virulence in ERIC I allows for the broader dissemination of spores?***

**Author response:** Thank you so much for this insightful suggestion. The text has been revised to clarify the mechanistic link between lower virulence and enhanced spore dissemination. The text now reads:

**Page 3-4, Line 107-111:** “*ERIC I strains typically take longer to kill infected larvae, allowing infected larvae to survive until cell capping, which reduces the efficiency of hygienic removal by nurse bees; as a result, infected larvae remain within capped cells, allowing P. larvae to complete sporulation and produce high numbers of infectious spores, and contribute to disease progression within and between colonies.”*

***L96 – If ERIC type II leads to quicker larval death, how would this accelerate the AFB outbreak? Do you mean the spread or the scale of affected larvae? Please clarify.***

**Author response:** Thank you very much for this question. We revised this sentence by changing the word outbreak. In fact, we refer to the acceleration as a rate of colony deterioration rather than increased transmission efficiency. The sentence now reads:

**Page 4, Line 113-115:** *“This results in the accumulation of infected and diseased brood before effective hygienic removal can occur, leading to accelerated brood loss and earlier colony collapse.”*

***L112 – For the entire set of phenotypic experiments, did you use any controls apart from your 6 tested isolates? I am referring to the ATCC 9545 strain and/or other P. larvae strain that have been described previously in the research literature? If no, please clearly indicate it.***

**Author response:** Thank you very much for this clarification. No additional reference or control strains were included in the phenotypic experiments. This has now been clearly stated in the revised manuscript, in the methods section as follows:

**Page 16, Line 556-559:** “*For phenotypic characterisation, only the six* P. larvae *isolates were used to assess strain-specific variability within each ERIC type, and no additional reference or control strains were included.”*

***L170, 346 – resistance to what?***

**Author response:** Thank you. We meant to refer to antibiotic resistance. This has now been clearly stated in the revised manuscript.

**Page 6, Line 197:** “*Several antibiotic resistance-associated loci were specific to ERIC I genomes”.*

***L171 – please indicate percent identity and coverage for the antibiotic-resistant genes; also, provide a comment on their functionality to put in the context.***

**Author response:** Thank you for this suggestion. We have now added the percent identity and sequence coverage values for the identified antibiotic resistance genes in the revised manuscript. In addition, we included a brief interpretation of their functional relevance, clarifying their potential role in conferring resistance based on the annotated gene functions and available database evidence.

**Page 6-7, Line 197-222:** “*One notable region comprises an IS630-associated tetB-like cassette, encoding a major facilitator superfamily (MFS) transporter was identified (100% coverage, 58.2% amino acid identity to UniProtKB homologues). The locus is flanked by a transposase and a split putative ABC transporter showing 100% coverage and 85% and 59% identity to homologues from Paenibacillus riograndensis and P. graminis, respectively.*

*A catB-like chloramphenicol resistance gene was identified in ERIC I genomes, encoding a chloramphenicol acetyltransferase with 100% coverage and 97.2% amino acid identity to a homologue from* Bacillus salipaludis*. A vanA-like determinant, annotated by Comprehensive Antibiotic Resistance Database (CARD) as vanF (100% coverage, 97.9% identity), was detected in all ERIC isolates except ERIC II strain 7851 and is associated with reduced glycopeptide resistance in* Paenibacillus popilliae *via D-Ala–D-Lac peptidoglycan synthesis.*

*ERIC I genomes also carried an arsenic resistance cassette together with additional detoxification loci, including a phenolic acid decarboxylase system and genes associated with membrane stress responses and efflux mechanisms.*

*ERIC II genomes, by comparison, carried a transposon-associated resistance island containing an additional copy of (sugE) together with ykkD, as annotated by Prokka. BLASTP showed SugE is homologous to the guanidinium efflux protein GdnC from* Bacillus licheniformis *(93% coverage, 43.4% identity), while YkkD is similar to GdnD from* Bacillus subtilis *(100% coverage, 32.7% identity). UniProtKB further identified YkkD as a QacE-family small multidrug resistance transporter, with 100% identity to* P. larvae *and 84.6% identity to* Paenibacillus agilis*. These proteins likely form membrane efflux systems contributing to tolerance of quaternary ammonium compounds and other toxic metabolites”.*

***L195 – please provide the total number of isolates analyzed.***

**Author response:** Thank you for this suggestion. The total number of isolates analysed has now been clearly stated in the revised manuscript, and the sentence has been updated as follows:

**Page 7, Line 240-242:** “*To explore the evolutionary relationship among the selected ERIC I and ERIC II P. larvae isolates, we performed a phylogenetic analysis using a total of 782 publicly available P. larvae genomes along with our six newly sequenced genomes”.*

***L203 – could you please add a measure for the “not too closely related”-ness? Eg branch length?***

**Author response:** Thank you very much for this suggestion. As suggested, we have now quantified the genetic distinctness of the selected isolates using median patristic distances. Specifically, we calculated the median patristic distance between the selected isolates and all isolates of the corresponding lineage and compared this to the median within-lineage distance across the full phylogeny. We have revised the paragraph as follows:

**Page 7-8, Line 249-257:** *“(…) were distributed across multiple sub-clades within the ERIC I and ERIC II lineages rather than forming a closely related cluster. Median patristic distances between the selected isolates and all isolates of the corresponding lineage exceeded within-lineage distances across the full phylogeny (ERIC I: 3.7×10⁻⁴ vs 2.7×10⁻⁴ substitutions per site; ERIC II: 9.3×10⁻⁵ vs 7.2×10⁻⁵ substitutions per site), demonstrating that the selected isolates are genetically diverse members of both major P. larvae lineages.”*

***L210-211 – Were there any indication of differences in the metabolism genes? If so and they are relevant to this experiment, please add them in here.***

**Author response:** This is something we did not investigate in detail, primarily because we realized it was not straightforward to identify with sufficient confidence all the metabolic genes, beyond those in the conserved central carbon metabolism and TCA cycle. To uncover all metabolic pathways, a more comprehensive genome analysis would be required, which would include gene similarity searches for many genes that have not been annotated with function. We feel that this is beyond the scope of this study, but it is something we would like to investigate in future work in a more dedicated study comparing metabolism between the various ERIC types.

***L230 – I assume it was done in broth?***

**Author response:** Yes, we used “*liquid MYPGP medium”* for the morphological characteristics of ERIC I and ERIC II strains.

***L240 – could you please provide an absolute measure too?***

**Author response:** Absolute cell length measurements have now been included alongside the relative fold difference to improve clarity, and the sentence has been updated as follows:

**Page 8, Line 290-292:** “*Consistent with our qualitative observations, ERIC I strains measured approximately 18-23 µm in length during log-phase growth and were at least threefold longer than ERIC II strains, which measured approximately 5-8 µm”.*

***L252 – it is a comparison between transformed vs not transformed or in between transformed ERIC I and ERIC II?***

**Author response:** The comparison was performed between the transformed ERIC I and ERIC II datasets, rather than between the transformed and non-transformed data. However, based on the comment of another reviewer, we have added the quantitative comparison of cellular morphology between the transformed and non-transformed strains for both genotypes. This has now been clarified in the revised manuscript as follows:

***Page 9, Line 306-311:*** *“Moreover, comparison of the transformed and non-transformed strains showed that the presence of the plasmid resulted in a significant increase in cell length in both ERIC I and ERIC II strains during logarithmic growth (Fig. S3E). We also found that during the stationary phase, cell length increased significantly in ERIC I strains carrying the plasmid, whereas no significant differences were observed in ERIC II strains compared with their plasmid-free counterparts (Fig. S3F).”*

***L270 – broth sporulation efficiency***

**Author response:** Yes, it was broth sporulation efficiency, and we have clarified this point in the revised manuscript as follows:

**Page 9, Line 318-320:** *“To comprehensively assess the sporulation efficiency of ERIC I and ERIC II strains, we examined sporulation efficiency in both liquid broth and solid MYPGP media, supplemented with glucose, fructose, and trehalose.”*

***L292 – run-on sentence?***

**Author response:** Thank you so much for pointing this out. The sentence has been revised for improved clarity and readability as follows:

**Page 10, Line 345-347:** “*Although the selected ERIC II strain appeared slower to develop phase-bright spores than the ERIC I strain, both strains progressed through the different stages of sporulation at a comparable rate.”*

***L300, 617 – up to date, there was no indication of work on P. larvae spore morphology and anatomy. Please explain how you decided/confirmed P. larvae spore anatomy and cite appropriate references.***

**Author response**: Due to the limited availability of species-specific ultrastructural descriptions for Paenibacillus larvae, spore layer identification in this study was based on the conserved and well-established structural organisation of endospores in Firmicutes, particularly Bacillus subtilis, which is widely used as a reference model for interpreting spore ultrastructure, along with closely related species such as *P. polymyxa*. Appropriate references have been included, and this clarification has been incorporated into both the manuscript text and the methodology section as follows:

**Page 10, Line 358-361: *“****We therefore examined spore envelope structure in three ERIC I and three ERIC II strains using TEM, based on the established spore architecture of endospore-forming bacteria such as* Bacillus subtilis and *Paenibacillus polymyxa [23,24]*.”

**Page 19, Line 684-685: "***Peptidoglycan thickness in TEM images was measured as the distance between the spore core and coat, corresponding to the germ cell wall and cortex layers, as described in the canonical endospore architecture of Firmicutes [23,24]”.*

***L307 – since there is a great degree of variation in spore composition and anatomy, why didn't you choose any other Paenibacillus spp. spores for a reference? Please advise.***

**Author response**: Thank you very much for this suggestion. In addition to *Bacillus subtilis,* we also used published spore architecture from closely related *Paenibacillus* species, particularly *Paenibacillus polymyxa,* as references for the identification of spore structural layers observed by TEM. Although this was not explicitly mentioned in the first draft of the manuscript, we have now revised the text to clarify this point as follows:

**Page 10, Line 354-357: *“****We therefore examined spore envelope structure in three ERIC I and three ERIC II strains using TEM, based on the established spore architecture of endospore-forming bacteria such as* Bacillus subtilis and *Paenibacillus polymyxa*.”

***L317 – you start this section indicating that germination happens in the honey bee larvae, yet there is no indication that your experiment was conducted in vitro, e.g., in the broth rather than in honey bee larvae; please rewrite accordingly.***

**Author response**: We agree that the original wording could incorrectly suggest that germination was directly assessed within honeybee larvae. To avoid this ambiguity, we have revised the text as follows to clearly indicate that the germination dynamics of ERIC I and ERIC II strains were evaluated using an *in vitro* germination assay.

**Page 11, Line 379-381: “***To test this hypothesis, we examined the germination dynamics of ERIC I and ERIC II strains using an in vitro broth-based germination assay.”*

***L329 – provide absolute value? Eg how much fold/times higher germination than in media without germinants?***

**Author response**: We agree that reporting the absolute germination values provides better clarity and allows a more direct comparison between conditions. We have therefore revised the text as follows:

**Page 11, Line 385-388: “***As expected, the germination efficiency was highest in media supplemented with both uric acid and L-tyrosine (27-38%), whereas lower germination efficiencies were observed in media containing only one germinant (17-22%) or no germinants (15-18%)
(Fig. 5B).”*

***L412-415 – looks like this finding is or might be limited by the number of genomes analysed in the study.***

**Author response**: We agree that the relatively limited number of genomes analysed could influence this finding. However, the analysed strains were broadly distributed across the phylogenetic tree and therefore represent diversity.

***L449 – please provide a reference or rewrite as this might mislead readers re: spore persistence.***

**Author response**: We agree that the original wording may have overstated the relationship between peptidoglycan thickness and environmental persistence. We have therefore revised the sentence and added references describing the role of spore peptidoglycan in spore resistance as follows:

**Page 14, Line 490-492: “***An overall thicker peptidoglycan layer in ERIC I spores may contribute to increased resistance to adverse conditions, as spore peptidoglycan and cortex structure are known to play important roles in spore resistance properties [51].”*

***L476 – please add a couple of sentences/a small paragraph for limitations of this study; in my opinion, this would allow readers to appreciate your findings and put them in the right context.***

**Author response**: Thank you for this suggestion. A small paragraph outlining the limitations of this study has now been added as follows:

***Page 15, Line 521-527: Limitations of the study***

*“While this study advances our understanding of P. larvae biology, a key limitation is that the findings may not fully reflect the biology of this pathogen during in vivo infection. Laboratory-cultivated organisms often exhibit different behaviours compared with those grown within the host or under host-mimicking conditions. Therefore, it is essential to examine and compare P. larvae growth, sporulation, and germination under both in vitro and in vivo conditions to identify characteristics specific to each of these conditions.”*

***L507 – What exact saccharides did you use?***

**Author response**: We used Glucose and this has now been specified in the method section as follows:

***Page 16, Line 561-563: “****Briefly,* P. larvae *strains were streaked onto MYPGP agar plates containing 12% glucose and supplemented with nalidixic acid (20 µg/mL) and pipemidic acid (10 µg/mL)”.*

***L511-512 – In between original recovery culture on MYPGP, with nalidixic and pipemidic acids, did you culture P. larvae on non-selective standard MYPGP or other media, e.g., CBA, to prevent media-induced changes in phenotype? Just to be clear: if you are culturing P. larvae on 12% saccharide media and then transferring it directly to broth with glucose/fructose or trehalose, wouldn't you expect increased sugar consumption (a potentiation of the growth effect), since the isolate has been pre-adapted on the saccharide MYPGP agar?***

**Author response**: Thank you for this question. We did not transfer *P. larvae* directly from the saccharide-containing MYPGP agar to the sugar-supplemented broth. Instead, after recovery on MYPGP agar containing nalidixic and pipemidic acids, we subcultured the isolates once on non-selective brain heart infusion (BHI) agar before inoculating them into broth supplemented with glucose, fructose, or trehalose. BHI agar does not contain added saccharides, so this intermediate passage prevents media-induced pre-adaptation to sugar. We have now clarified this procedure in the Methods section of the revised manuscript as follows:

***Page 16, Line 566-571: “****Isolates recovered on MYPGP agar supplemented with nalidixic acid and pipemidic acid were subcultured once on non-selective BHI agar to eliminate potential sugar pre-adaptation effects. Following this intermediate passage, single colonies were selected and used to assess the effect of different saccharides on bacterial growth by culturing the strains overnight in MYPGP broth supplemented with either glucose, fructose, or trehalose, and adjusted to an OD₆₀₀ of 0.05.”*

***L514 – I assume that 24 growth was monitored in sugar-supplemented MYPGP broth; however, it is not clear, please indicate***

**Author response**: We agree that the original wording did not clearly indicate that growth monitoring was performed in the sugar-supplemented MYPGP broth. We have revised the sentence to clarify this point as follows:

***Page 16, Line 571-572: “****Bacterial growth in the respective sugar-supplemented MYPGP broths was subsequently monitored over a 24 h period using a Tecan microplate reader.”*

***L526-527 – If the “slight modification of the protocol” is not included elsewhere, please add it in the manuscript, as it is essential to your work.***

**Author response**: We agree that the modifications to the published protocol should be explicitly described. We have therefore revised this part in the methods section to clarify the modification as follows:

***Page 17, Line 586-588: “****Briefly, different chloramphenicol concentrations were evaluated for strain selection, and 30 µg/mL was determined to provide optimal selection efficiency for the strains used in this study, compared with the 5 µg/ml concentration described in the original protocol [18].”*

***L530 – I see results for the ICE, prophages, and antibiotic resistance genes in the “Results” section, yet I couldn't find anything in the M&M section. Please add information on your in silico analysis for the above-mentioned features if you haven`t done so.***

**Author response**: Thank you very much for this observation. We now include detailed information on the *in silico* analysis of ICEs (integrative and conjugative elements), prophages, and antibiotic resistance genes in the Materials and Methods section as follows:

**Page 17, Line 602-605: *“****The assembled genomes were consequently annotated using the Prokka annotation pipeline (v1.13) [67] and screened for AMR-associated genes against CARD database (entries from April 2023) [68] using ABRicate. PhiSpy (v4.2.21) [69] was used to aid prophage region identification.”*

***L555 – Please indicate that you analyzed X number of P. larvae isolates in the first sentence.***

**Author response**: We agree that the total number of analysed isolates should be clearly stated at the beginning of the section. We have therefore revised the first sentence to indicate that phylogenetic and co-conservation analyses were performed using six newly sequenced P. larvae isolates together with 782 publicly available genomes. The text now reads:

**Page 18, Line 619-622:** *“For phylogenetic and co-conservation analyses, we used six newly sequenced P. larvae isolates obtained from different geographical locations, representing a balanced subset of ERIC I and ERIC II genotypes (three isolates per ERIC type), together with 782 publicly available P. larvae complete genomes retrieved from the NCBI database in September 2025.”*

***L578 – your references don`t match the text, please fix it.***

**Author response**: We are sorry for this oversight and thank you for pointing it out. We have carefully checked and corrected the reference as follows:

**Page 24, Reference 61*.*** *“Mahdi OS, Fisher NA. Growth and laboratory maintenance of Paenibacillus larvae. Current Protocols in Microbiology. 2018 Jan;48(1):9E-1.”*

***L590 – In my personal experience, when harvesting P. larvae spores, sometimes it is hard to properly homogenize scraped colonies with vortexing only. Were there any other homogenization methods you used? If so, please indicate; if not, then you are a lucky one!***

**Author response**: Yes, we also experienced the same *P. larvae* spores can sometimes be challenging when using vortexing alone. For this reason, in our protocol, after scraping the colonies from solid media, samples were first washed three times with 10 mL of PBS before undergoing four additional washes with sterile, cold deionised water, with repeated vortexing and centrifugation. We have now clarified this procedure in the revised manuscript. The text now reads:

***Page 19, Line 655-658:*** *“Scraped colonies from solid media were first washed three times with 10 mL PBS, followed by four washes with 15 mL sterile, cold deionised water by repeated vortexing and centrifugation (3000 × g, 15 min, 4 °C) in 15 mL of sterile, cold deionised water using 50 mL centrifuge tubes.”*

***L635 – Were there any other methods employed to verify germination success, e.g., direct microscopy of aliquots, DPA (dipicolinic acid) test, etc.? if so, please indicate.***

**Author response**: We are looking to establish protocols for measurements of DPA and time-lapse imaging of germinating spores in the laboratory. But in this work, no additional methods were employed to independently verify germination success in this study.

***Figure 4A. Please indicate what is the stratified layer of the spore between OC and IC? (marked in red)***

**Author response**: The apparent stratified layer between the outer coat (OC) and inner coat (IC) resulted from a slight displacement of the label in the original figure. This has now been corrected to accurately indicate the OC and IC regions - no additional layer exists between them.

Previous version

Revised version

***Reviewer #2****: The manuscript entitled “Comparative genomics and phenotypic 1 divergence of ERIC I and ERIC II genotypes of Paenibacillus larvae, the causative agent of American foulbrood disease” reports the genomic and phenotypic characterization of three strains of P. larvae genotype ERIC I and three of P. larvae genotype ERIC II aimed at explaining the difference in invasiveness and pathogenicity of these two most widespread variants of the AFB causative agent. The article is written in perfect UK English and the presentation is very good. However, in my opinion, the aim is not well stated at the end of the introduction and the abstract conclusion does not render the value of the study. Its novelty mainly lies in showing the different physiological and genetic features that influence environmental adaptability and pathogenicity of the two P. larvae most widespread genotypes and this must be better specified and the study originality should be better underlined in comparison with previous investigations. Defining core, shell and cloud genomes on just six strains is too limited so I suggest to enlarge the analysis by including some of the publicly available genome sequences, chosen according to valid criteria, to establish the distinctive genetic features of the two genotypes on a wider basis.*

We thank the reviewer for their feedback. In addition to the specific comments raised, we have also improved the aims in the introduction and made the value of the study clearer in the abstract.

**Reviewer #2:**

***Lines 53-56: this conclusion is unclear. That found at lines 491-493 is more appropriate.***

**Author response:** Thank you very much for this suggestion and we have now modified the abstract to state the conclusion per the recommendation. This sentence has been updated as follows:

***Page 2, Line 53-55: “****Collectively, our findings improve understanding of P. larvae biology and provide a foundation for developing genotype-specific disease management strategies for AFB.”*

***Table 1 could be omitted, giving the information in the text. In particular, the continent column is superfluous***

**Author response:** Thank you for this suggestion. We agree that th and have removed it from Table 1. In addition, the relevant information from the table has now been incorporated into the main text in the Results section.

**Page 4, Line 140-146:** “*ERIC I strains (7841, 7845, and 7856) showed genome sizes ranging from 4.10 to 4.16 Mb with GC contents of approximately 44.0%. In contrast, ERIC II strains (7842, 7851, and 7853) had smaller genome sizes, ranging from 3.69 to 3.74 Mb, and slightly higher GC contents of approximately 44.5%. Assembly metrics also differed between the two groups, with ERIC I strains comprising 134-178 contigs and N50 values ranging from 61,840 to 80,118 bp, whereas ERIC II strains contained 297-311 contigs and showed lower N50 values ranging from 25,137 to 25,910 bp.”*

***Lines 164-166: please, spell out Cmr and CRISPR-Cas here at first occurrence also here besides in Materials and Methods***

**Author response:** Thank you so much for this suggestion. We have now spelt out both Cmr and CRISPR-Cas at their first occurrence in the manuscript for improved clarity. This sentence has been updated as follows:

**Page 6, Line 189-191:** *Both genotypes encode a Type III-B Clustered Regularly Interspaced Short Palindromic Repeats (CRISPR)-CRISPR-associated (Cas) module. However, ERIC II genomes along with ERIC I-7845 harbour a more complete CRISPR module RAMP (Cmr)-associated cluster (…)*

***Lines 180-182: please spell out ETX and SCIFF***

**Author response:** We have now spelt out both ETX and SCIFF at their first occurrence in the manuscript to improve clarity. This sentence has been updated as follows:

**Page 7, Line 224-230:** “*ERIC I genomes possess an expanded repertoire of toxin- and competition-related regions, including an IS256-associated pagA/Tc-like toxin cluster, an epsilon toxin (ETX)-family toxin-like gene, a large Rhs polymorphic toxin cassette, and two bacteriocin loci, one of which is associated with a two-component regulatory system. In addition, a Six Cysteines in Forty-Five Residues (SCIFF) peptide module and a skf-associated cluster further contribute to the competition-related gene repertoire. These loci were consistently detected across all ERIC I genomes.”*

**Discussion: please, shorten the parts that repeat too much the results, e.g. Lines 344-351; 354-362**

**Author response:** Thank you for this insight – we agree there is too much repetition of the results. Therefore, we have removed these sections of text altogether.

**Page 11-12, Line 399-403:** “*Consistent with previous reports [26,27], our comparative genomic analysis of the* P. larvae *strains revealed clear distinctions between ERIC I and ERIC II groups, alongside substantial conservation of core genetic content (Fig. 1A&B, S1A). Analyses of their pan-genomes corroborated these genomic trends. Most genes were shared, emphasising vertical inheritance of the core genome, while accessory genes contributed to ERIC-specific differences.”*

***Lines 491-493: this should be also stated at the end of the abstract***

**Author response:** As above - we have now modified the abstract to state this conclusion per the recommendation. This sentence has been updated as follows:

***Page 2, Line 53-55: “****Collectively, our findings improve understanding of P. larvae biology and provide a foundation for developing genotype-specific disease management strategies for AFB.”*

***Line 501: please, cite by the first author’s name before the citation number***

**Author response:** We have revised the sentence to include the first author’s name before the citation number, as suggested.

**Page 16, Line 553-555: *“****The genotypes of the selected strains were previously characterised using the multilocus sequence typing (MLST) scheme described in Morrissey et al. [60].”*

***Line 502: please, add the location for FERA***

**Author response:** We have now added the location of the UK Food and Environment Research Agency (FERA) in the revised manuscript. This sentence has been updated as follows:

**Page 16, Line 555-559: *“****The isolates were obtained from the UK Food and Environment Research Agency (FERA), York, UK, and are listed in Table 1.”*

***Line 504: please, specify the types of methods***

**Author response:** We have revised the subsection title from “General methods” to “Culture of *P. larvae*” to better reflect the content of the section.

**Page 16, Line 561: “**Culture of *P. larvae”*

***Reviewer #3:*** *In this manuscript, Hossain et  al. present a genetic and phenotypic comparison of Paenibacillus larvae ERIC I and ERIC II strains. The authors investigate differences in genomic features, growth characteristics, and cellular morphology. Overall, the methodologies employed are appropriate and robust, and generally the conclusions drawn are well supported by the data presented, with one exception, which is noted below. This is an interesting manuscript and makes a valuable contribution to understanding P. larvae at both the genetic and phenotypic levels. I have a small number of minor comments, which are outlined below.*

We thank the reviewer for their positive feedback and for recognising the value of our work. Their minor comments are addressed below

***There appear to be several issues with referencing throughout the manuscript. This may reflect problems with the referencing software used or in some instances the most appropriate primary literature is not cited. All references should be carefully checked for accuracy, relevance, and correctness.***

**Author response:** Thank you so much for pointing this out. We have gone through the text and have improved the referencing, including referring to the primary source rather than reviews.

***Specific examples include: Citation 1: The primary literature from which this information is derived should be cited rather than a secondary source.***

**Author response:** Thank you for this comment. We have carefully checked the citation and confirm that the information is referenced using the original primary literature source. The updated reference is as follows:

**Page 22, Reference 1:** “*Hung KL, Kingston JM, Albrecht M, Holway DA, Kohn JR. The worldwide importance of honeybees as pollinators in natural habitats. Proceedings of the Royal Society B: Biological Sciences. 2018 Jan 1;285(1870).”*

***Citation 3: This reference relates to bacteriophage use as a biocontrol strategy and does not support statements regarding declining honeybee populations.***

**Author response:** We are sorry for this and agree that Citation 3 was not appropriate for this statement. We have removed this citation and replaced it with a more relevant reference that directly supports the discussion of honeybee declines and their contributing factors.

**Page 22, Reference 3: “***Goulson D, Nicholls E, Botías C, Rotheray EL. Bee declines driven by combined stress from parasites, pesticides, and lack of flowers. Science. 2015 Mar 27;347(6229):1255957.”*

***Citation 4: This reference addresses the honeybee microbiota more broadly and is not specific to Paenibacillus larvae.***

**Author response:** Thank you for pointing this out. The citation has been revised to include a more suitable reference that specifically supports the statement regarding American foulbrood as a major brood disease of honeybees caused by *Paenibacillus larvae.*

**Page 22, Reference 4: “***Alqurneh M, Bergmann NT, Nairoukh I, Hamdan AJ, Kaatz HH. High prevalence of Paenibacillus larvae, the pathogenic agent of American foulbrood disease, in Palestinian honey bee colonies. Veterinary Research Communications. 2026 Apr;50(2):111.”*

***Citation 66: This reference concerns the SPAdes assembler and does not relate to spore production.***

**Author response:** We are sorry for this oversight. It may have occurred due to the referencing software. We have now carefully checked every citation, and citation 66 has been corrected and replaced with the appropriate reference.

**Page 26, Reference 66: *“****Bankevich A, Nurk S, Antipov D, Gurevich AA, Dvorkin M, Kulikov AS, Lesin VM, Nikolenko SI, Pham S, Prjibelski AD, Pyshkin AV. SPAdes: a new genome assembly algorithm and its applications to single-cell sequencing. Journal of computational biology. 2012 May 1;19(5):455-77.”*

***For clarity and consistency, the terminology used to describe American foulbrood should be standardised throughout the manuscript. Variants currently used include “American Foul Brood,” “American Foulbrood,” and “American foulbrood”; the authors should select one form and apply it consistently.***

**Author response:** We agree that consistent terminology is important for clarity. The terminology has now been standardised throughout the manuscript, and the term “*American foulbrood”* has been used consistently.

***Line 90: A manuscript about the discovery of ERIC V is cited, despite ERIC V not being mentioned in the text.***

**Author response:** Thank you for pointing this out. We have now revised the text to include ERIC V, consistent with the cited reference, and it now reads as follows:

**Page 3, Line 104-106: “**P. larvae *is known to be an extremely variable pathogen, and through ERIC (Enterobacterial Repetitive Intergenic Consensus) typing, different* P. larvae *genotypes have been identified, namely ERIC I, II, III, IV and V [14,15].”*

***The manuscript states that insertion of plasmid pAD43-25 causes a significant increase in cell length; however, the data supporting this claim are unclear. While figures are provided for cells with and without the plasmid, no direct comparative analysis is presented. Additional explanation and/or quantitative analysis is required to substantiate this observation.***

**Author response:** Thank you very much for this suggestion. We agree that a clearer quantitative comparison is needed to support the claim regarding cell length differences. We have now performed an additional quantitative analysis comparing wild-type (WT) and GFP-expressing cells (WT vs. GFP) in terms of cell length. We have added new figures (Figure S3E&F) showing the distribution of cell lengths and a statistical comparison. The corresponding text has also been updated to clearly describe this finding as follows:

**Page 9, Line 306-311: “***Moreover, comparison of the transformed and non-transformed strains showed that the presence of the plasmid resulted in a significant increase in cell length in both ERIC I and ERIC II strains during logarithmic growth (Fig. S3E). We also found that during the stationary phase, cell length increased significantly in ERIC I strains carrying the plasmid, whereas no significant differences were observed in ERIC II strains compared with their plasmid-free counterparts (Fig. S3F).”*

Length (µm)

WT

GFP

WT

GFP

Length (µm)

**E**

ERIC I

ERIC II

**F**

Length (µm)

WT

GFP

Length (µm)

WT

GFP

ERIC I

ERIC II

***Finally, Supplementary Figure 6 is not referenced in the main text. All supplementary figures should be clearly cited and integrated into the narrative where relevant.***

**Author response:** Thank you so much for this observation. Supplementary Figure 6 has now been cited in the main text and integrated into the relevant section of the manuscript as follows:

**Page 11, Line 379-383: “ (…)** we examined the germination dynamics of ERIC I and ERIC II strains using an in vitro broth-*based germination assay. Purified spores were incubated for 18 h in MYPGP medium alone, or in MYPGP medium supplemented with the known germinants: uric acid, L-tyrosine or a combination of both (Fig. S6)”*

***Part II – Major Issues: Key Experiments Required for Acceptance*** *Please use this section to detail the key new experiments or modifications of existing experiments that should be absolutely required to validate study conclusions.

Generally, there should be no more than 3 such required experiments or major modifications for a "Major Revision" recommendation. If more than 3 experiments are necessary to validate the study conclusions, then you are encouraged to recommend "Reject".*

***Reviewer #1:*** *No major issues have been identified.*

***Reviewer #2:*** *I suggest to enlarge the comparative genome analysis by including some of the publicly available genome sequences, chosen according to valid criteria, to establish the distinctive genetic features of the two genotypes on a wider basis*

**Author response:** We have discussed these analyses with the editor, and with their agreement, we think these additional analyses are not required because based on the phylogenetic diversity of the strains we tested, our data have already established distinctive genetic features of ERIC I and ERIC II. In other words, our isolates are diverse enough to conclude that their characteristics are representative genetic features of ERIC I and ERIC II.

**Reviewer #3**: (No Response)

**Part III – Minor Issues: Editorial and Data Presentation Modifications**
Please use this section for editorial suggestions as well as relatively minor modifications of existing data that would enhance clarity.
