# Supplementary material for: Comparative genomics and phenotypic divergence of ERIC I and ERIC II genotypes of Paenibacillus larvae, the causative agent of American Foulbrood disease
Source: PLoS Pathog. 2026 Aug 13;22(8):e1014507. doi: 10.1371/journal.ppat.1014507 (PMC13497268; doi:10.1371/journal.ppat.1014507)
Supplement: Peer review history

# Peer review: Decision Letter 0

**DOI:** 10.1371/journal.ppat.1014507.r001

**Authors:** Alice Prince, William Navarre

3 May 2026

PPATHOGENS-D-26-00634

Comparative genomics and phenotypic divergence of ERIC I and ERIC II genotypes of Paenibacillus larvae, the causative agent of American foulbrood disease

PLOS Pathogens

Dear Dr. Rodrigues,

Thank you for submitting your manuscript to PLOS Pathogens. After careful consideration, we feel that it has merit but does not fully meet PLOS Pathogens's publication criteria as it currently stands. Therefore, we invite you to submit a revised version of the manuscript that addresses the points raised during the review process.

Please submit your revised manuscript by Jul 02 2026 11:59PM. If you will need more time than this to complete your revisions, please reply to this message or contact the journal office at plospathogens@plos.org. When you're ready to submit your revision, log on to https://www.editorialmanager.com/ppathogens/ and select the 'Submissions Needing Revision' folder to locate your manuscript file.

Please include the following items when submitting your revised manuscript:

* A letter that responds to each point raised by the editor and reviewer(s). You should upload this letter as a separate file labeled 'Response to Reviewers'. This file does not need to include responses to any formatting updates and technical items listed in the 'Journal Requirements' section below.

* A marked-up copy of your manuscript that highlights changes made to the original version. You should upload this as a separate file labeled 'Revised Manuscript with Track Changes'.

* An unmarked version of your revised paper without tracked changes. You should upload this as a separate file labeled 'Manuscript'.

If you would like to make changes to your financial disclosure, competing interests statement, or data availability statement, please make these updates within the submission form at the time of resubmission. Guidelines for resubmitting your figure files are available below the reviewer comments at the end of this letter.

We look forward to receiving your revised manuscript.

Kind regards,

William Navarre

Academic Editor

PLOS Pathogens

Alice Prince

Section Editor

PLOS Pathogens

Sumita Bhaduri-McIntosh

Editor-in-Chief

PLOS Pathogens

orcid.org/0000-0003-2946-9497

Michael Malim

Editor-in-Chief

PLOS Pathogens

orcid.org/0000-0002-7699-2064

**Additional Editor Comments :**

Your manuscript was reviewed by three experts. All felt the work was solid but that the writing was unclear in several places (see the extensive edits from reviewer 1). Please address these concerns.

**Journal Requirements:**

1) Please provide an Author Summary. This should appear in your manuscript between the Abstract (if applicable) and the Introduction, and should be 150-200 words long. The aim should be to make your findings accessible to a wide audience that includes both scientists and non-scientists. Sample summaries can be found on our website under Submission Guidelines:

https://journals.plos.org/plospathogens/s/submission-guidelines#loc-parts-of-a-submission

2) Please upload all main figures as separate Figure files in .tif or .eps format. For more information about how to convert and format your figure files please see our guidelines:

https://journals.plos.org/plospathogens/s/figures

3) Tables should not be uploaded as individual files. Please remove these files and include the Tables in your manuscript file as editable, cell-based objects. For more information about how to format tables, see our guidelines:

https://journals.plos.org/plospathogens/s/tables

4) We have noticed that you have uploaded Supporting Information files, but you have not included a list of legends. Please add a full list of legends for your Supporting Information files after the references list.

5) Please ensure that the funders and grant numbers match between the Financial Disclosure field and the Funding Information tab in your submission form. Note that the funders must be provided in the same order in both places as well. Currently, the order of the funders is different in both places.

Note: If the reviewer comments include a recommendation to cite specific previously published works, please review and evaluate these publications to determine whether they are relevant and should be cited. There is no requirement to cite these works unless the editor has indicated otherwise.

**Reviewers' Comments:**

**Comments to the Authors:**

**Please note that one review is uploaded as an attachment.**

Reviewer's Responses to Questions

**Part I - Summary**

Please use this section to discuss strengths/weaknesses of study, novelty/significance, general execution and scholarship.

Reviewer #1: Presented for the peer-review paper titled “Comparative genomics and phenotypic divergence of ERIC I and ERIC II genotypes of Paenibacillus larvae, the causative agent of American foulbrood disease” submitted to Plos Pathogens provides novel information on genetic and phenotypic differences between the two major genotypes of P. larvae - ERIC type I and ERIC type II. The manuscript provides a strong and reliable experimental design for future in-depth in silico-in vitro-in vivo studies in P. larvae and highlights the importance of research in bacterial pathogenesis, especially for the American Foulbrood.

This manuscript is a well-written, well-characterized study that uses WGS and phenotypic testing to significantly advance understanding of the inherited genetic differences in P. larvae and their role in subsequent AFB pathogenesis. This has great potential to evolve into a more comprehensive, larger study to uncover previously undescribed aspects of AFB pathogenesis and subsequently inform evidence-based countermeasures in the field. In contrast, a couple of aspects of this manuscript need improvement and/or clarification to ensure that the work presented was conducted properly. Additionally, there are several minor mistakes/ incorrect statements that need to be changed and supported with proper citation, as they might mislead readers. Lastly, the wording of some sentences is confusing and needs to be fixed for clarity and readability.

Reviewer #2: The manuscript entitled “Comparative genomics and phenotypic 1 divergence of ERIC I and ERIC II genotypes of Paenibacillus larvae, the causative agent of American foulbrood disease” reports the genomic and phenotypic characterization of three strains of P. larvae genotype ERIC I and three of P. larvae genotype ERIC II aimed at explaining the difference in invasiveness and pathogenicity of these two most widespread variants of the AFB causative agent. The article is written in perfect UK English and the presentation is very good. However, in my opinion, the aim is not well stated at the end of the introduction and the abstract conclusion does not render the value of the study. Its novelty mainly lies in showing the different physiological and genetic features that influence environmental adaptability and pathogenicity of the two P. larvae most widespread genotypes and this must be better specified and the study originality should be better underlined in comparison with previous investigations. Defining core, shell and cloud genomes on just six strains is too limited so I suggest to enlarge the analysis by including some of the publicly available genome sequences, chosen according to valid criteria, to establish the distinctive genetic features of the two genotypes on a wider basis.

Reviewer #3: In this manuscript, Hossain et  al. present a genetic and phenotypic comparison of Paenibacillus larvae ERIC I and ERIC II strains. The authors investigate differences in genomic features, growth characteristics, and cellular morphology. Overall, the methodologies employed are appropriate and robust, and generally the conclusions drawn are well supported by the data presented, with one exception, which is noted below. This is an interesting manuscript and makes a valuable contribution to understanding P. larvae at both the genetic and phenotypic levels. I have a small number of minor comments, which are outlined below.

**********

**Part II – Major Issues: Key Experiments Required for Acceptance**

Please use this section to detail the key new experiments or modifications of existing experiments that should be absolutely required to validate study conclusions.

Generally, there should be no more than 3 such required experiments or major modifications for a "Major Revision" recommendation. If more than 3 experiments are necessary to validate the study conclusions, then you are encouraged to recommend "Reject".

Reviewer #1: No major issues have been identified.

Reviewer #2: I suggest to enlarge the comparative genome analysis by including some of the publicly available genome sequences, chosen according to valid criteria, to establish the distinctive genetic features of the two genotypes on a wider basis

Reviewer #3: (No Response)

**********

**Part III – Minor Issues: Editorial and Data Presentation Modifications**

Please use this section for editorial suggestions as well as relatively minor modifications of existing data that would enhance clarity.

Reviewer #1: (Limit 20000 Characters)

See line-by-line comments below.

NCBI SRA project data – will be released by the time the paper is published?

L40 – Foulbrood is a one-word term as it is the name of a disease rather than a characteristic of brood.

L60 – I would say European honey bee since you are referring to the A. mellifera.

L68 – sentence needs to be rewritten to more clearly indicate that AFB is a disease and P. larvae is its causal agent.

L76-77 – Please provide a reference to why early age larval susceptibility makes disease prevention particularly challenging?

L77 – please indicate the sequence of AFB pathogenesis eg “ingestion-proliferation in the midgut-invasion through midgut epithelium – septiceamia”, as the sentence implies that proliferation in the midgut and hemolymph happens in parallel.

L90 – please include ERIC type V as well as per Beims et al.

L92-93 – Please elaborate on how lower virulence in ERIC I allows for the broader dissemination of spores?

L96 – If ERIC type II leads to quicker larval death, how would this accelerate the AFB outbreak? Do you mean the spread or the scale of affected larvae? Please clarify.

L112 – For the entire set of phenotypic experiments, did you use any controls apart from your 6 tested isolates? I am referring to the ATCC 9545 strain and/or other P. larvae strain that have been described previously in the research literature? If no, please clearly indicate it.

L170, 346 – resistance to what?

L171 – please indicate percent identity and coverage for the antibiotic-resistant genes; also, provide a comment on their functionality to put in the context.

L195 – please provide the total number of isolates analyzed.

L203 – could you please add a measure for the “not too closely related”-ness? Eg branch length?

L210-211 – were there any indication of differences in the metabolism genes? If so and they are relevant to this experiment, please add them in here.

L230 – I assume it was done in broth?

L240 – could you please provide an absolute measure too?

L252 – it is a comparison between transformed vs not transformed or in between transformed ERIC I and ERIC II?

L270 – broth sporulation efficiency

L292 – run-on sentence?

L300, 617 – up to date, there was no indication of works on P. larvae spore morphology and anatomy. Please explain how you decided/confirmed P. larvae spore anatomy and cite appropriate references.

L307 – since there is a great degree of variation in spore composition and anatomy, why didn't you choose any other Paenibacillus spp. spores for a reference? Please advise.

L317 – you start this section indicating that germination happens in the honey bee larvae, yet there is no indication that your experiment was conducted in vitro, e.g., in the broth rather than in honey bee larvae; please rewrite accordingly.

L329 – provide absolute value? Eg how much fold/times higher germination than in media without germinants?

L412-415 – looks like this finding is or might be limited by the number of genomes analyzed in the study.

L449 – please provide a reference or rewrite as this might mislead readers re: spore persistence.

L476 – please add a couple of sentences/a small paragraph for limitations of this study; in my opinion, this would allow readers to appreciate your findings and put them in the right context.

L507 – What exact saccharides did you use?

L511-512 – In between original recovery culture on MYPGP, with nalidixic and pipemidic acids, did you culture P. larvae on non-selective standard MYPGP or other media, e.g., CBA, to prevent media-induced changes in phenotype? Just to be clear: if you are culturing P. larvae on 12% saccharide media and then transferring it directly to broth with glucose/fructose or trehalose, wouldn't you expect increased sugar consumption (a potentiation of the growth effect), since the isolate has been pre-adapted on the saccharide MYPGP agar?

L514 – I assume that 24 growth was monitored in sugar-supplemented MYPGP broth; however, it is not clear, please indicate

L526-527 – If the “slight modification of the protocol” is not included elsewhere, please add it in the manuscript, as it is essential to your work.

L530 – I see results for the ICE, prophages, and antibiotic resistance genes in the “Results” section, yet I couldn't find anything in the M&M section. Please add information on your in silico analysis for the above-mentioned features if you haven`t done so.

L555 – Please indicate that you analyzed X number of P. larvae isolates in the first sentence.

L578 – your references don`t match to the text, please fix it.

L590 – In my personal experience, when harvesting P. larvae spores, sometimes it is hard to properly homogenize scraped colonies with vortexing only. Were there any other homogenization methods you used? If so, please indicate; if not, then you are a lucky one!

L635 – Were there any other methods employed to verify germination success, e.g., direct microscopy of aliquots, DPA (dipicolinic acid) test, etc.? if so, please indicate.

Figure 4A. Please indicate what is the stratified layer of the spore between OC and IC? (marked in red)

Reviewer #2: Specific remarks

Lines 53-56: this conclusion is unclear. That found at lines 491-493 is more appropriate

Table 1 could be omitted giving the information in the text. In particular, the continent column is superfluous

Lines 164-166: please, spell out Cmr and CRISPR-Cas here at first occurrence also here besides in Materials and Methods

Lines 180-182: please, spell out ETX and SCIFF

Discussion: please, shorten the parts that repeat too much the results, e.g. Lines 344-351; 354-362

Lines 491-493: this should be also stated at the end of the abstract

Line 501: please, cite by the first author’s name before the citation number

Line 502: please, add the location for FERA

Line 504: please, specify the types of methods

Reviewer #3: There appear to be several issues with referencing throughout the manuscript. This may reflect problems with the referencing software used or in some instances the most appropriate primary literature is not cited. All references should be carefully checked for accuracy, relevance, and correctness.

Specific examples include:

Citation 1: The primary literature from which this information is derived should be cited rather than a secondary source.

Citation 3: This reference relates to bacteriophage use as a biocontrol strategy and does not support statements regarding declining honeybee populations.

Citation 4: This reference addresses the honeybee microbiota more broadly and is not specific to Paenibacillus larvae.

Citation 66: This reference concerns the SPAdes assembler and does not relate to spore production.

For clarity and consistency, the terminology used to describe American foulbrood should be standardised throughout the manuscript. Variants currently used include “American Foul Brood,” “American Foulbrood,” and “American foulbrood”; the authors should select one form and apply it consistently.

Line 90: A manuscript about the discovery of ERIC V is cited, despite ERIC V not being mentioned in the text.

The manuscript states that insertion of plasmid pAD43-25 causes a significant increase in cell length; however, the data supporting this claim are unclear. While figures are provided for cells with and without the plasmid, no direct comparative analysis is presented. Additional explanation and/or quantitative analysis is required to substantiate this observation.

Finally, Supplementary Figure 6 is not referenced in the main text. All supplementary figures should be clearly cited and integrated into the narrative where relevant.

**********

PLOS authors have the option to publish the peer review history of their article (what does this mean?). If published, this will include your full peer review and any attached files.

If you choose “no”, your identity will remain anonymous but your review may still be made public.

**Do you want your identity to be public for this peer review?**  For information about this choice, including consent withdrawal, please see our Privacy Policy.

Reviewer #1: No

Reviewer #2: No

Reviewer #3: No

[NOTE: If reviewer comments were submitted as an attachment file, they will be attached to this email and accessible via the submission site. Please log into your account, locate the manuscript record, and check for the action link "View Attachments". If this link does not appear, there are no attachment files.]

**Figure resubmission:**

**Reproducibility:**

To enhance the reproducibility of your results, we recommend that authors of applicable studies deposit laboratory protocols in protocols.io, where a protocol can be assigned its own identifier (DOI) such that it can be cited independently in the future. Additionally, PLOS ONE offers an option to publish peer-reviewed clinical study protocols. Read more information on sharing protocols at https://plos.org/protocols?utm_medium=editorial-email&utm_source=authorletters&utm_campaign=protocols

Attachment Submitted filename: 2026.04.05_review_PLOS.docx

# Author comment: Author response to Decision Letter 1

**DOI:** 10.1371/journal.ppat.1014507.r002

15 Jun 2026

Attachment Submitted filename: Final-Response to reviewers v1.docx

# Peer review: Decision Letter 1

**DOI:** 10.1371/journal.ppat.1014507.r003

**Authors:** Alice Prince, William Navarre

27 Jul 2026

Dear Dr. Rodrigues,

We are pleased to inform you that your manuscript 'Comparative genomics and phenotypic divergence of ERIC I and ERIC II genotypes of Paenibacillus larvae, the causative agent of American Foulbrood disease' has been provisionally accepted for publication in PLOS Pathogens.

Before your manuscript can be formally accepted you will need to complete some formatting changes, which you will receive in a follow up email. A member of our team will be in touch with a set of requests.

Please note that your manuscript will not be scheduled for publication until you have made the required changes, so a swift response is appreciated.

IMPORTANT: The editorial review process is now complete. PLOS will only permit corrections to spelling, formatting or significant scientific errors from this point onwards. Requests for major changes, or any which affect the scientific understanding of your work, will cause delays to the publication date of your manuscript.

Should you, your institution's press office or the journal office choose to press release your paper, you will automatically be opted out of early publication. We ask that you notify us now if you or your institution is planning to press release the article. All press must be co-ordinated with PLOS.

Thank you again for supporting Open Access publishing; we are looking forward to publishing your work in PLOS Pathogens.

Best regards,

William Navarre

Academic Editor

PLOS Pathogens

Alice Prince

Section Editor

PLOS Pathogens

Sumita Bhaduri-McIntosh

Editor-in-Chief

PLOS Pathogens

orcid.org/0000-0003-2946-9497

Michael Malim

Editor-in-Chief

PLOS Pathogens

orcid.org/0000-0002-7699-2064

***********************************************************

Thank you for taking the reviewer's comments seriously and incorporating their suggestions into your revised manuscript. All three reviewers are satisfied with the changes that have been made.

Reviewer Comments (if any, and for reference):

Reviewer's Responses to Questions

**Part I - Summary**

Please use this section to discuss strengths/weaknesses of study, novelty/significance, general execution and scholarship.

Reviewer #1: Presented for the peer-review paper titled “Comparative genomics and phenotypic divergence of ERIC I and ERIC II genotypes of Paenibacillus larvae, the causative agent of American foulbrood disease” submitted to Plos Pathogens provides novel information on genetic and phenotypic differences between the two major genotypes of P. larvae - ERIC type I and ERIC type II. The manuscript provides a strong and reliable experimental design for future in-depth in silico-in vitro-in vivo studies in P. larvae and highlights the importance of research in bacterial pathogenesis, especially for the American Foulbrood.

I am satisfied with the provided corrections in the manuscript and recommend it`s acceptance.

Reviewer #2: The manuscript entitled "Comparative genomics and phenotypic divergence of ERIC I and ERIC II genotypes of Paenibacillus larvae, the causative agent of American Foulbrood disease" presents an original genetic and phenotypic characterization of the two most widespread lineages of the honeybee pathogen Paenibacillus larvae known as ERIC types I and II. Some aspects that determine the different behaviour of these two P. larvae variants such as sugar sources utilization, sporulation and germination dynamics and spore ultrastructure were defined with the aim to explain the different pathogenicity features and find strategies to limit their capacity to cause the disease. The main weakness of the study is the lack of in vivo proof.

Reviewer #3: The authors have put in substantial effort to address the concerns of the reviewers, and I thank them for their hard work. I have reviewed the revised version and am satisfied that my concerns have been addressed. The manuscript has been markedly improved, and I have no further comments.

I am happy to recommend acceptance of the manuscript in its current form.

**********

**Part II – Major Issues: Key Experiments Required for Acceptance**

Please use this section to detail the key new experiments or modifications of existing experiments that should be absolutely required to validate study conclusions.

Generally, there should be no more than 3 such required experiments or major modifications for a "Major Revision" recommendation. If more than 3 experiments are necessary to validate the study conclusions, then you are encouraged to recommend "Reject".

Reviewer #1: none

Reviewer #2: No major issues found

Reviewer #3: (No Response)

**********

**Part III – Minor Issues: Editorial and Data Presentation Modifications**

Please use this section for editorial suggestions as well as relatively minor modifications of existing data that would enhance clarity.

Reviewer #1: L90-91 – please add more detail on exactly why “this early susceptibility … makes disease prevention particularly challenging”. First, the susceptibility isn't “early” – it is age-dependent; second, there are several bodies of work – including Goodman et al. from New Zealand – that describe multiple prevention methods; please revise the statement.

L107– 115: please double-check the information here. To the best of my knowledge, the difference in virulence is inversely proportionate to the degree of clinical sign manifestation of AFB in the honey bee colony exactly because of hygienic behavior of the bees; e.g. if ERIC type II has higher virulence – larvae die faster – bees remove dead larvae, and it is harder to diagnose visually, which contributes to the spread of ERIC type II-born AFB. In my experience, both ERIC type I and ERIC type II produce several million spores; therefore, this factor might not be the primary driver of AFB progression and transmission between hives.

Reviewer #2: No need for changes in presentation

Reviewer #3: (No Response)

**********

PLOS authors have the option to publish the peer review history of their article (what does this mean?). If published, this will include your full peer review and any attached files.

If you choose “no”, your identity will remain anonymous but your review may still be made public.

**Do you want your identity to be public for this peer review?**  For information about this choice, including consent withdrawal, please see our Privacy Policy.

Reviewer #1: No

Reviewer #2: No

Reviewer #3: No

# Editor report: Acceptance letter

**DOI:** 10.1371/journal.ppat.1014507.r004

**Authors:** Alice Prince, William Navarre

Dear Dr. Rodrigues,

We are delighted to inform you that your manuscript, "Comparative genomics and phenotypic divergence of ERIC I and ERIC II genotypes of Paenibacillus larvae, the causative agent of American Foulbrood disease," has been formally accepted for publication in PLOS Pathogens.

We have now passed your article onto the PLOS Production Department who will complete the rest of the pre-publication process. All authors will receive a confirmation email upon publication.

The corresponding author will soon be receiving a typeset proof for review, to ensure errors have not been introduced during production. Please review the PDF proof of your manuscript carefully, as this is the last chance to correct any scientific or type-setting errors. Please note that major changes, or those which affect the scientific understanding of the work, will likely cause delays to the publication date of your manuscript. Note: Proofs for Front Matter articles (Pearls, Reviews, Opinions, etc...) are generated on a different schedule and may not be made available as quickly.

Soon after your final files are uploaded, the early version of your manuscript, if you opted to have an early version of your article, will be published online. The date of the early version will be your article's publication date. The final article will be published to the same URL, and all versions of the paper will be accessible to readers.

For Research Articles, you will receive an invoice from PLOS for your publication fee after your manuscript has reached the completed accept phase. If you receive an email requesting payment before acceptance or for any other service, this may be a phishing scheme. Learn how to identify phishing emails and protect your accounts at https://explore.plos.org/phishing.

Thank you again for supporting open-access publishing; we are looking forward to publishing your work in PLOS Pathogens.

Best regards,

Sumita Bhaduri-McIntosh

Editor-in-Chief

PLOS Pathogens

orcid.org/0000-0003-2946-9497

Michael Malim

Editor-in-Chief

PLOS Pathogens

orcid.org/0000-0002-7699-2064
